# Supplementary material for: Sex differences in the association between area deprivation and generalised anxiety disorder: British population study
Source: BMJ Open. 2017 May 4;7(5):e013590. doi: 10.1136/bmjopen-2016-013590 (PMC5777465; doi:10.1136/bmjopen-2016-013590)
Supplement: supplementary appendix [file bmjopen-2016-013590supp001.pdf]

Appendix I: Characteristics of participants who consented (n=30,445) and refused (n=43,452) to take part in the EPIC-Norfolk cohort study

Percentage (number)		
**Characteristic**	**Consented**	**Did not consent**
**Age**		
<50	27.5 (8366)	33.7 (14647)
50-60	30.3 (9230)	29.5 (12819)
60-70	32.5 (9879)	27.4 (11898)
>70	9.8 (2970)	9.4 (4088)
**Sex**		
Female	55.0 (16744)	49.0 (21296)
Male	45.0 (13701)	51.0 (22156)
