# Supplementary material for: Sex differences in the association between area deprivation and generalised anxiety disorder: British population study
Source: BMJ Open. 2017 May 4;7(5):e013590. doi: 10.1136/bmjopen-2016-013590 (PMC5777465; doi:10.1136/bmjopen-2016-013590)
Supplement: Author's manuscript [file bmjopen-2016-013590.draft_revisions.pdf]

BMJ Open

Area deprivation and generalised anxiety disorder: British population study

Journal:	*BMJ Open*
Manuscript ID	bmjopen-2016-013590
Article Type:	Research
Date Submitted by the Author:	22-Jul-2016
Complete List of Authors:	Remes, Olivia; University of Cambridge, Department of Public Health and Primary Care<br>Wainwright, Nicholas; University of Cambridge, UK, Department of Public Health and Primary Care<br>Surtees, Paul; University of Cambridge, Department of Public Health and Primary Care<br>LaFortune, Louise; University of Cambridge, Department of Public Health and Primary Care<br>Khaw, Kay-Tee; University of Cambridge, Department of Public Health and Primary Care<br>Brayne, Carol; University of Cambridge, Department of Public Health and Primary Care
**Primary Subject Heading**:	Mental health
Secondary Subject Heading:	Public health, Epidemiology
Keywords:	EPIDEMIOLOGY, MENTAL HEALTH, Anxiety disorders < PSYCHIATRY, PUBLIC HEALTH

SCHOLARONE™
Manuscripts

Area deprivation and generalised anxiety disorder: British population study

Olivia Remes[1], Nick Wainwright[2], Paul Surtees[1], Louise Lafortune[1], Kay-Tee Khaw[3], Carol Brayne[1]

[1]Department of Public Health and Primary Care, University of Cambridge, Forvie Site, Robinson Way, Cambridge, UK CB2 0SR

[2](Retired) Department of Public Health and Primary Care, University of Cambridge, 2 Worts' Causeway, Cambridge, UK CB1 8RN

[3]Department of Public Health and Primary Care, University of Cambridge, 2 Worts' Causeway, Cambridge, UK CB1 8RN

Word count: 3392; Figures: 1; Tables: 3; References: 42; Supplementary files: 0

Correspondence to: Olivia Remes or260@medschl.cam.ac.uk, telephone: 0777674550

For peer review only

ABSTRACT

OBJECTIVE

To determine whether area-level deprivation is associated with generalised anxiety disorder over and above individual-level circumstances, and whether this association is different between British women and men.

DESIGN

Large, population study.

SETTING

UK population-based cohort.

PARTICIPANTS

30,445 people from the general population aged 40 years and older and living in England consented to participate at study baseline, and of these, 21,000 participants completed a structured health and lifestyle questionnaire used to capture generalised anxiety disorder. Area deprivation was measured in 1991 using Census data, and generalised anxiety disorder was assessed in 1996-2000. 10,275 women and 8,219 men had complete data on all covariates.

MAIN OUTCOME MEASURE

Past-year generalised anxiety disorder defined according to the Diagnostic and Statistical Manual of Mental Disorders, fourth edition (DSM-IV).

RESULTS

In this study, 2.5% (261/10,275) of women and 1.8% (145/8,219) of men had generalised anxiety disorder. Women living in the most deprived areas were over 60% more likely to develop anxiety than those living in areas that were not deprived (OR=1.63, 95%CI: 1.21 to 2.21; p=0.001), but this association between deprivation and generalised anxiety disorder was not apparent in men (OR=1.13, 95% CI: 0.72 to 1.77; p=0.598).

CONCLUSION

The absolute numbers of people living in deprived conditions are large worldwide. This, combined with a growing mental health burden means that the findings obtained in this study have been and continue to be highly relevant. The World Health Organisation has emphasised the need to reduce social and health inequalities. Our findings provide a strong evidence base to this call, showing that the environment needs to be taken into account when developing mental health policy; gender is clearly an important factor when it comes to assessing the impacts of the environment, and promoting good mental health.

Key words: Anxiety, anxiety disorders, risk factors, gender

For peer review only

Strengths and limitations of this study

- We used a large, population-based sample of middle- and older-aged adults and adequately adjusted for a range of important confounders.
- We used a structured, self-reported questionnaire to assess presence of past-year GAD, and participants were followed for a long period of time.
- We used validated and reliable measures of area deprivation.
- Those who participated in this study were somewhat less deprived and healthier than individuals living in other parts of England; therefore, our results may not generalize to people living in extremely deprived circumstances.

For peer review only

INTRODUCTION

Generalized anxiety disorder (GAD) is a common and persistent disorder, and is associated with increased risk for disability and suicide.[1–4] GAD can lead to serious impairment in social and occupational functioning, and once it develops, it increases the risk for major depression, substance misuse, and serious physical medical conditions.[4–7] This disorder has a chronic course and is difficult to treat.[4] Consequently, it is important that its risk factors are identified for prevention and targeted intervention.

Few studies have assessed the risk factors of GAD, therefore, evidence-based information is scarce. The studies that have been undertaken have focused on characteristics measured at the level of the individual, such as, personal income and education[8, 9, 10], demographics[11, 12], and family history of psychopathology.[12] However, previous research has shown that the living context, such as deprivation, can have profound effects on health, independent of personal characteristics. Many previous studies conducted in western countries have shown that living in areas characterized by high income inequality can lead to significantly increased risks for serious medical conditions and mortality.[13, 14] Whether area deprivation can be used to predict generalized anxiety disorder (GAD) is unknown.

In this population-based, cohort study, we examine the association between area deprivation and generalized anxiety disorder, while controlling for a number of confounders, including previous medical conditions, major depressive disorder, and

For peer review only

sociodemographic factors. Results are presented separately for women and men. All participants recruited through general-practice registers and who completed a baseline health questionnaire were eligible to be included in our study; those who completed a baseline health check and a psychosocial questionnaire during follow-up were eligible to be included in our analysis. Our hypothesis was that deprivation would be associated with an increased risk of developing generalized anxiety disorder in women and men.

For peer review only

METHODS

Data were drawn from the European Prospective Investigation of Cancer (EPIC)-Norfolk, whose design and study methods have been described in detail elsewhere.[15] In brief, a prospective population-based cohort of 30,445 participants ages 40 to 74 years were recruited by post between 1993 and 1997 through general practice age-sex registers in the city of Norwich and the surrounding small towns and rural areas. At baseline (1993-97), 30,445 participants consented to join the study and completed a postal Health and Lifestyle (HLQ) questionnaire that captured information on sociodemographics, including age, sex, marital status, highest educational attainment, socio-economic status, and self-reported physician diagnoses of physical diseases. Using participants' postal codes, two measures of area deprivation were derived based on the 1991 Census and the 2001 Census. During follow-up, participants completed self-reported postal questionnaires provided they: 1) were still alive, 2) did not ask to be removed from the study's mailing list, and 3) had a valid mailing address.

During 1996-2000, 20,921 participants completed a structured, psychosocial Health and Life Experiences (HLEQ) questionnaire. During this time, an assessment of generalized anxiety disorder (GAD) and major depressive disorder (MDD) was made according to the Diagnostic and Statistical Manual of Mental Disorders, fourth edition. (Figure 1).[16] Using the HLEQ questionnaire, disability measures based on the SF-36 were also derived.[17]

For peer review only

Dependent variable

The primary outcome in this study was past-year GAD. The self-reported HLEQ questionnaire captured the onset and offset timings of episodes of past-year GAD.[16] Past-year GAD consisted of at least one episode that had offset within 12 months of administration of the HLEQ. DSM-IV GAD was diagnosed if participants reported having uncontrollable, excessive worry for six months or longer on most days than not that resulted in disability or impairment. In addition, at least three of the following symptoms needed to have been present: restlessness, irritability, muscle tension, fatigue, trouble concentrating because of worry, mind going blank, trouble falling asleep, trouble staying asleep, and feeling keyed up or on edge.

Individual-level measures (potential confounders)

Individual-level measures included age, sex, social class, marital status, and educational level. Social class was derived using the Computer-Assisted Standard Occupational Coding[18] and categorized as follows: I (professionals), II (managerial and technical occupations), III non-manual and III manual (skilled workers), IV (partly skilled workers), and V (unskilled manual workers). To assign social class to men and women, the male partner's current or past occupation was used. If this information was not available, the female partner's occupation was used. If the social class from either partner was unavailable, then it was coded as missing. Marital status was categorized into three groups: married, single (or never married), and others (widowed, divorced, separated).

For peer review only

Educational attainment was categorized into high (vocational or formal qualifications at the A- or O-level or degree-level qualifications) vs. low (no formal qualifications).

Individual-level health status was assessed through the construction of a variable capturing major prevalent physical diseases associated with anxiety. This was based on HLQ questions asking participants: "Has the doctor ever told you that you have any of the following?", followed by a list of options, such as allergies, asthma, cancer, stroke, heart attack, diabetes, thyroid conditions, etc. To determine disability levels, we used the physical component summary score (PCS) of the Medical Outcomes Study 36-Item Short Form (SF-36), a widely-used, validated self-assessment tool. A score of 100 represents no disability and 0 represents a high level of disability.[17] PCS scores were dichotomized above and below the median.

All of these individual-level variables were regarded as potential confounders and selected based on the literature and their association with anxiety and deprivation.

Area-level measure (exposure variable)

To examine area deprivation, we used two of the most commonly-used measures of area deprivation in the UK: the Townsend Index[19] and the Index of Multiple Deprivation[20]. The Townsend index was based on 1991 Census data and the IMD was based on the 2001 Census (constructed using an improved methodology - this methodology was not available for earlier Census cycles.). These two measures are

For peer review only

180 therefore composite measures from individuals completing Census data. Participants were linked to the Census using their postal codes, and deprivation scores based on the Townsend index and the IMD were created. The Townsend index is measured at the level of the enumeration district and the IMD at the level of the Lower layer Super Output Area (LSOA), a small, homogeneous geographical region containing approximately 1,500 people. The Townsend index captures information on unemployment, non-car ownership, non-home ownership, and household overcrowding. The IMD is an aggregated measure of income, employment, health, education, housing and services, crime, and the living environment.

For peer review only

Statistical analysis

Characteristics of the participants were compared by GAD status. We used correlated data analysis to assess the association between individual- and area-level risk factors of GAD. A population-average model was constructed, which accounted for the potential correlation introduced by the clustering of individuals within LSOAs. To estimate the population-average effect of the risk factors of interest on past-year GAD, we used generalized estimating equations. As past-year GAD represents a binary outcome (yes/no) and the intra-cluster correlation is assumed to be equal, GEE with a logit link and an exchangeable correlation structure was used. Adjusted odds ratios (OR) and 95% confidence intervals based on robust standard errors were estimated. Standard multivariate logistic regression was also conducted and compared to the findings based on GEE.

Individual-level measures consisted of demographic and socio-economic status variables, whereas area-level measures comprised the Townsend index and the IMD. Townsend index scores were used to create a dichotomous variable, with 0 as the cut-point (representing the national average). IMD scores were categorized into quartiles from least to highest deprivation.

Analyses were conducted separately for men and women. First, unadjusted effect estimates were determined. Next, models were constructed that adjusted for 1) age, social class, educational attainment; then for 2) age, social class, educational

For peer review

attainment, lifetime history of MDD; and finally for 3) age, social class, educational attainment, lifetime history of MDD, physical diseases, and disability level. Age was first assessed as a categorical variable, and subsequently divided into 10-year bands. Models were constructed for participants with complete measurements on all covariates. Unless stated otherwise, all analyses used the Townsend index as the measure of deprivation. Each model was built on people for whom we had complete measures on all covariates. The brackets show the reference categories that were used for each categorical variable when it was entered in the models - deprivation: no [ref] vs. yes; GAD: no [ref] vs. yes; education: high [ref] vs. low; marital status: married [ref], single, others; social class: non-manual [ref] vs. manual; lifetime MDD: no [ref] vs. yes; prevalent physical disease: no [ref] vs. yes; disability level: low [ref] vs. high. These reference categories were based on the literature. Choosing other groupings for the potential confounders would not have changed the results. It was not possible to group the GAD variable otherwise, and area deprivation was analysed in accordance with the literature.

Finally, analyses were run with pure GAD as the outcome, in which past-year MDD was excluded. All models used two-sided statistical tests and a p-value of <0.05 was considered statistically significant. Analyses were implemented in SAS Version 9.3 (SAS Institute, Cary, NC).

To arrive at the study size, we went through the following steps: of the 30,445 who completed the baseline HLQ, we retained those participants who completed the HLEQ

For peer review only

(20,921), and of these, we kept those people with complete data on all covariates (18,494). (Figure 1)

Patient involvement

There were no patients involved in the development of the research question and outcome measures, the design of the study, or the recruitment to and conduct of the study.

For peer review only

RESULTS

77,630 people from general practices in Norfolk were invited to take part in the study, and of these, 30,445 consented. Of the 30,445 people recruited at baseline, 20,921 completed the HLEQ during follow-up. Of those who completed the HLEQ, 18,494 (88.4%) were available for analysis in this study, because they had data on all covariates. The number of missing observations for each covariate were: 9 for education, 47 for marital status, 497 for GAD, 468 for MDD, 458 for social class, 34 for the Index of Multiple Deprivation, 75 for the Townsend index, and 1386 for the SF-36. Participants were followed between 1993 and 2000 (7 years).

The study sample consisted of a total of 10,275 women and 8,219 men over the age of 40. Table 1 shows the distribution of individual- and area-level characteristics by past-year GAD.

For peer review only

Table 1: Distribution of characteristics for women (n=10,275) and men (n=8,219) who completed the HLEQ questionnaire in the EPIC-Norfolk cohort

	Women		Men	
**Characteristic**	**Number with characteristic**	**Percentage and number with past-year GAD**	**Number with characteristic**	**Percentage and number with past-year GAD**
**Socio-demographics**				
**Age (years)**				
<50	1444	3.7 (54)	961	3.2 (31)
50-60	3693	3.2 (119)	2645	2.4 (63)
60-70	3167	1.9 (61)	2739	1.2 (33)
>70	1971	1.4 (27)	1874	1.0 (18)
**Education**‡				
Low	4030	2.1 (83)	2363	1.7 (39)
High	6245	2.9 (178)	5856	1.8 (106)
**Marital status**				
Single	414	3.1 (13)	302	4.0 (12)
Married	7714	2.4 (183)	7221	1.5 (111)
Other*	2147	3.0 (65)	696	3.2 (22)
**Social class**¥				
Manual	3820	2.3 (89)	3281	1.7 (55)
Non-manual	6455	2.7 (172)	4938	1.8 (90)
**Townsend index**				
**Deprivation**				
Yes (>0)	1636	3.9 (64)	1237	2.3 (28)
No (<=0)	8639	2.3 (197)	6982	1.7 (117)
**Health status**				
**Prevalent physical disease**+				
Yes	5660	3.1 (174)	3836	2.2 (86)
No	4615	1.9 (87)	4383	1.4 (59)
**Disability level**				
High¶	5258	3.3 (172)	4009	2.6 (104)
Low	5017	1.8 (89)	4210	0.97 (41)
**Lifetime MDD**				
Yes	1926	8.7 (167)	934	10.0 (93)
No	8349	1.1 (94)	7285	0.7 (52)

For peer review only

+ Prevalent physical disease: respiratory disease (asthma and bronchitis), allergies (allergies and hay fever), stroke, heart attack, cancer, diabetes, thyroid conditions, arthritis

¥ Manual: skilled manual, semi-skilled, non-skilled; non-manual: professionals, managerial, skilled non-manual

‡ High education: O-level, A-level, degree; low education: refers to no education

* Other: divorced, separated, widowed

¶ Above the median PCS value of 50.6

For peer review only

The overall prevalence of past-year GAD was 2.5% (261/10275) for women and 1.8% (145/8219) for men. Women and men with GAD were more likely to be younger, of higher educational attainment, single, in non-manual occupations, with prevalent physical diseases, higher levels of disability, and major depressive disorder (MDD).

Findings from the correlated data analysis showed that the risk of GAD in women living in the most deprived areas was over 70% higher than in those living in the least deprived areas, even after adjusting for age and socio-economic status (OR=1.77, 95% CI: 1.33–2.36) (table 2).

For peer review only

Table 2: Odds ratios for past-year GAD according to individual- and area-level characteristics for women (n=10,275) who completed the HLEQ questionnaire in the EPIC-Norfolk cohort

	Odds ratios (OR) and 95% CI				
**Characteristic**	**Unadjusted**	**Model A[1]**	**Model B[2]**	**Model C[3]**	**P-value for Model C**
**Socio-demographics**					
**Age**					
(per 10 years)	0.65 (0.56–0.74)	0.63 (0.54–0.73)	0.73 (0.62–0.85)	0.66 (0.56–0.77)	<0.0001
**Education‡**					
Low	0.72 (0.55–0.93)	0.85 (0.64–1.12)	0.90 (0.68–1.20)	0.90 (0.68–1.20)	0.475
High	1.00	1.00	1.00	1.00	
**Marital status**					
Single	1.33 (0.75–2.36)	1.31 (0.73–2.36)	1.36 (0.74–2.50)	1.34 (0.73–2.47)	0.348
Married	1.00	1.00	1.00	1.00	
Other*	1.28 (0.96–1.71)	1.48 (1.09–2.00)	1.09 (0.80–1.48)	1.07 (0.79–1.46)	0.671
**Social class¥**					
Manual	0.87 (0.67–1.13)	0.89 (0.68–1.17)	0.89 (0.68–1.18)	0.85 (0.64–1.13)	0.271
Non-manual	1.00	1.00	1.00	1.00	
**Townsend index**					
**Deprivation**					
Yes (>0)	1.74 (1.31–2.32)	1.77 (1.33–2.36)	1.65 (1.23–2.22)	1.63 (1.21–2.21)	0.001
No (<=0)	1.00	1.00	1.00	1.00	
**Health status**					
**Lifetime MDD**					
Yes	8.34 (6.44–10.79)		7.55 (5.78–9.86)	7.00 (5.34–9.17)	<0.0001
No	1.00		1.00	1.00	
**Prevalent physical disease+**					
Yes	1.65 (1.27–2.14)			1.43 (1.09–1.88)	0.011
No	1.00			1.00	
**Disability level**					
High¶	1.87 (1.45–2.43)			1.88 (1.42–2.49)	<0.0001
Low	1.00			1.00	

1. Adjusted for age, SES (education, marital status, social class)

For peer review only

2. Adjusted for age, SES, lifetime MDD

3. Adjusted for age, SES, lifetime MDD, physical disease and disability

+ Prevalent physical disease: respiratory disease (asthma, bronchitis), allergies (allergies, hay fever), stroke, heart attack, cancer, diabetes, thyroid conditions, arthritis

¥ Manual: skilled manual, semi-skilled, non-skilled; non-manual: professionals, managerial, skilled non-manual

‡ High education: O-level, A-level, degree; low education: refers to no education

* Other: divorced, separated, widowed

¶ Above the median PCS value of 50.6

For peer review only

290 The OR reduced slightly after additionally controlling for major depressive disorder
291 (MDD) (OR=1.65, 95% CI: 1.23–2.22), but remained highly significant. A strong
292 association was present after further adjusting for prevalent physical diseases and
293 disability (OR=1.63, 95% CI: 1.21–2.21; p=0.001). In men, no association existed between
294 anxiety and area deprivation in both unadjusted and adjusted analyses (model C
295 OR=1.13, 95% CI: 0.72–1.77; p=0.598) (table 3).

For peer review only

Table 3: Odds ratios for past-year GAD according to individual- and area-level characteristics for men (n=8,219) who completed the HLEQ questionnaire in the EPIC-Norfolk cohort

	Odds ratios (OR) and 95% CI				
**Characteristic**	**Unadjusted**	**Model A[1]**	**Model B[2]**	**Model C[3]**	**P-value for Model C**
**Socio-demographics**					
**Age**					
(per 10 years)	0.59 (0.49–0.71)	0.58 (0.48–0.71)	0.63 (0.51–0.77)	0.52 (0.41–0.64)	<0.0001
**Education[‡]**					
Low	0.91 (0.63–1.32)	1.13 (0.75–1.70)	1.16 (0.78–1.74)	1.09 (0.73–1.63)	0.670
High	1.00	1.00	1.00	1.00	
**Marital status**					
Single	2.65 (1.44–4.86)	2.34 (1.26–4.36)	2.67 (1.39–5.10)	2.57 (1.32–5.01)	0.006
Married	1.00	1.00	1.00	1.00	
Other[*]	2.09 (1.31–3.33)	2.21 (1.39–3.52)	1.48 (0.90–2.44)	1.51 (0.91–2.51)	0.111
**Social class[¥]**					
Manual	0.92 (0.65–1.29)	0.83 (0.58–1.20)	0.84 (0.58–1.23)	0.74 (0.50–1.09)	0.125
Non-manual	1.00	1.00	1.00	1.00	
**Townsend index**					
**Deprivation**					
Yes (>0)	1.36 (0.90–2.06)	1.26 (0.82–1.94)	1.19 (0.76–1.85)	1.13 (0.72–1.77)	0.598
No (<=0)	1.00	1.00	1.00	1.00	
**Health status**					
**Life-time MDD**					
Yes	15.38 (10.87–21.76)		14.25 (9.97–20.37)	12.88 (8.99–18.46)	<0.0001
No	1.00		1.00	1.00	
**Prevalent physical disease[+]**					
Yes	1.68 (1.20–2.35)			1.53 (1.07–2.20)	0.021
No	1.00			1.00	
**Disability level**					
High[¶]	2.71 (1.88–3.90)			3.10 (2.13–4.51)	<0.0001
Low	1.00			1.00	

1. Adjusted for age, SES (education, marital status, social class)
2. Adjusted for age, SES, lifetime MDD
3. Adjusted for age, SES, lifetime MDD, physical diseases and disability

[†] Prevalent physical disease: respiratory disease (asthma, bronchitis), allergies (allergies, hay fever), stroke, heart attack, cancer, diabetes, thyroid conditions, arthritis

[¥] Manual: skilled manual, semi-skilled, non-skilled; non-manual: professionals, managerial, skilled non-manual

[‡] High education: O-level, A-level, degree; low education: refers to no education

[*] Other: divorced, separated, widowed

[¶] Above the median PCS value of 50.6

For peer review only

We had similar findings when logistic regression was used in these models instead of GEE, suggesting that the intra-class correlation is negligible (findings not shown).

When the Townsend index was replaced with quartiles of the IMD in the full multivariable model (C), there was an association between deprivation and GAD in women (quartile 4 vs quartile 1: fully adjusted OR=1.43, 95% CI: 1.01–2.02). The effect of the IMD was statistically significant (LR test, p-value = 0.01 on 3df). By contrast, no significant associations with GAD existed in men.

To assess whether deprivation was associated with *pure* past-year GAD in women, we excluded participants reporting past-year MDD (while controlling for all covariates in a fully-adjusted model). Deprivation continued to be strongly associated with past-year GAD (OR= 1.61, 95% CI: 1.06–2.43) (findings not shown). In men, the association was still statistically non-significant (OR=1.34, 95% CI: 0.73–2.47).

For peer review only

DISCUSSION

In this analysis of data from a population-based, cohort study we show, for the first time that area deprivation is significantly associated with increased risk for generalized anxiety disorder (GAD) in women, but not in men. The association in women was independent of characteristics measured at the level of the individual, including socio-demographics and major medical conditions. It is difficult to confirm causality between area deprivation and GAD; however, a rigorous analysis based on observational data is a reasonable method of examining this relationship. Our hypothesis has been supported for women, but not for men. Other research has shown that, compared to men, women living in areas characterized by socioeconomic disadvantage are at greater risk for depression and poor self-rated health. In the US, interventions that moved families to lower-poverty neighbourhoods substantially improved girls' mental health.[21] Our study is the first to investigate the effect of living in areas characterized by high deprivation on the risk of developing generalized anxiety disorder (GAD), and to examine this separately for women and men.

Potential mechanisms

The context as measured by Census composite deprivation indices appears to have a different relationship with the mental health of women and men, even after adjusting for individual socio-economic status, demographics, and other psychiatric and major medical conditions. Several mechanisms can account for this. Women perceive, relate to, and engage differently than men.[22, 23] Women spend more time in their local

For peer review only

environment, perhaps due to their greater uptake of part-time work and domestic or child-rearing duties.[24] Since they are more embedded in their local communities, they are also more likely to be exposed to the stress and strain that comes with living in deprived circumstances.[25, 26, 27] Exposure to stress has been associated with CNS dysfunction and HPA axis dysregulation, which have been implicated in the etiology of GAD.[28, 89] Women may also perceive the environment differently compared to men. Neighbourhood safety and fear of being assaulted appear to be much more of a concern for women. If women perceive their neighbourhood to be unsafe, they tend to restrict leisure activities, such as walking, and this can have further negative effects for their mental health.[26, 30] Men and women may also experience and manifest the effects of stress in different ways.[31] Women who are highly distressed tend to develop internalising disorders, while men are more prone to substance abuse and antisocial personality disorder.[32]

Another important mechanism that can be used to explain our findings is the link between deprivation, pollution, and anxiety. Poorer communities are disproportionately exposed to higher levels of pollution[33], and high pollution has been implicated in the development of systemic inflammation and anxiety[34]. Exposure to polycyclic aromatic hydrocarbons (a common air pollutant arising from motor vehicle emissions, oil and coal burning for heat and electricity, indoor space heaters and tobacco smoke) has been associated with mental health problems, including attention-deficit/hyperactivity disorder and cognitive delays.[33] The site of our study, Norfolk,

For peer review only

has been termed a ‘pollution hotspot’ in England and has recently been granted funding to reduce emissions from public transportation to meet EU targets on nitrogen dioxide.[35] These factors, combined with women’s greater vulnerability in developing anxiety disorders, make it very likely that living in deprivation leads to the development of GAD in this gender group. Deprivation can further have harmful consequences for future generations, thereby perpetuating the cycle of poverty and poor mental health.

Strengths and weaknesses and future research

This study reveals that anxiety in women is strongly linked with area disadvantage. It has several strengths. We had a large, population-based sample of middle- and older-aged adults and adequately adjusted for a range of possible confounders. We used a structured, self-reported questionnaire to assess presence of past-year GAD, and participants were followed for a long period of time. We overcome methodological limitations of previous studies by using two common measures of area deprivation capturing important features of the environment (such as access to health services and level of crime in an area) and these measures were available at two different levels – nevertheless, the associations remained the same. We also had a large list of self-reported physician diagnoses of chronic physical diseases that we used to establish medical histories. Despite this, the residual effect of diseases not captured by our study, but that are associated with GAD may be present. Past illness may have been underreported, which may have introduced measurement error and attenuated effect estimates towards the null. Participants were required to complete detailed dietary and

For peer review only

lifestyle questionnaires and undergo periodic health assessments. Because those who participated in EPIC-Norfolk were somewhat less deprived and healthier than individuals living in other parts of England[15], our results may not generalize to people living in extremely deprived circumstances.

Future research should consider assessing the risk of GAD in countries with high social and material inequalities, such as the United States, where the rates of anxiety are also some of the highest in the world.[36] Compared to the UK, the overall prevalence of GAD in the US is more than twice as high, and middle-aged people are most affected, with a prevalence of 7.7%.[37] It would be especially informative to repeat this study in less developed parts of the world, such as, India, where poverty is strongly linked to the development of mental disorders, and women's unequal status and social roles in society represent important additional issues.[38]

Implications for future generations and placing our research in context

The consequences of living in deprivation are far-reaching and can affect future generations. Repeated exposure to socioeconomic disadvantage in childhood is a consistent predictor of poor mental health in adolescence and young adulthood, particularly for young girls.[39] Since anxiety disorders tend to emerge in early adolescence, repeated exposure to socioeconomic disadvantage in childhood can increase the risk for more severe, early-onset forms of the disorder. Early-onset forms

For peer review only

are the most difficult to treat and have a poor prognosis.[40] Our study is the largest to date to examine the link between area deprivation and generalized anxiety disorder.

Our findings differ from the only other population-based, contextual study of generalized anxiety among men and women living in areas of low socioeconomic circumstances.[25] In this cross-sectional study, no association with anxiety was found; however, the measure of deprivation was based only on the local unemployment rate. Thus, the results are not directly comparable to ours. Further, the previous study used the Symptoms Checklist-90-Revised scale to measure *complaints* of generalized anxiety, yielding different estimates than ours. In contrast to the DSM-IV, the Symptoms Checklist-90-Revised scale did not base the definition of generalized anxiety around excessive, uncontrollable worry, which is the central, defining feature of GAD, and used a much shorter time frame to assess symptoms. We used a thorough assessment of DSM-IV GAD, which was measured in the past year. In contrast to the previous study, we also examined area deprivation using common, validated indices, covering a wide range of key domains relating to socio-economic disadvantage, such as, level of crime in an area and access to local health services. Studies assessing other health outcomes have suggested that the residential environment has a larger effect on women's health[23, 25], while individual-level factors relating to social status, such as, employment have the greatest impact on men's health.[22] Our study expands on previous research and is the first to investigate the effect of living in areas characterized

For peer review only

by high deprivation on the risk of developing generalized anxiety disorder (GAD), and to investigate this separately in women and men.

Interpretation

The absolute numbers of people living in deprived conditions are large worldwide. This, combined with a growing mental health burden means that the findings obtained in this study have been and continue to be highly relevant. The World Health Organisation[41] has emphasised the need to reduce social and health inequalities. Our findings provide a strong evidence base to this call, showing that “perhaps the most important risks to health are beyond people’s immediate control”[42] and that the environment needs to be taken into account when developing mental health policy. Additionally, gender is clearly an important factor when it comes to assessing the impacts of the environment, and promoting good mental health. Although it is difficult to confirm causality between area deprivation and anxiety, a rigorous analysis based on observational data is a reasonable method of examining this relationship.

For peer review only

Acknowledgements: OR received a PhD studentship from the National Institute for Health Research.

Competing interest: All authors have completed the ICMJE uniform disclosure form at www.icmje.org/coi_disclosure.pdf and declare: no support from any organisation for the submitted work; no financial relationships with any organizations that might have an interest in the submitted work in the previous three years; no other relationships or activities that could appear to have influenced the submitted work.

Funding: This work was supported by the Medical Research Council UK (grant number SP2024-0201 and SP2024-0204) and Cancer Research UK (grant number G9502233).

Author contributions: OR (corresponding author) had the idea for and conducted the analysis, and wrote the article, along with CB, KK, LL, PS, and NW. All authors provided feedback into the analysis and critically reviewed drafts of the manuscript. All authors have seen and approved the final version. The authors had full access to all the data in the study and take responsibility for the integrity of the data and the accuracy of the data analysis. OR acts as guarantor of the study.

Transparency declaration: OR affirms that the manuscript is an honest, accurate, and transparent account of the study being reported; that no important aspects of the study

For peer review only

have been omitted; and that any discrepancies from the study as planned have been explained.

Role of study sponsors and statement of independence: The funding sources had no role in the design and conduct of the study; collection, management, analysis, and interpretation of the data; and preparation, review, or approval of the manuscript.

Ethical approval: The study has ethics committee approval from Norfolk Ethics Committee (Rec Ref: 98CN01) and all participants gave informed consent.

Data sharing: No additional data available. Original dataset requests should be sent to the corresponding author. Please contact O Remes at or260@medschl.cam.ac.uk for questions about the statistical code.

Copyright: “The Corresponding Author has the right to grant on behalf of all authors and does grant on behalf of all authors, a worldwide licence to the Publishers and its licensees in perpetuity, in all forms, formats and media (whether known now or created in the future), to i) publish, reproduce, distribute, display and store the Contribution, ii) translate the Contribution into other languages, create adaptations, reprints, include within collections and create summaries, extracts and/or, abstracts of the Contribution, iii) create any other derivative work(s) based on the Contribution, iv) to exploit all subsidiary rights in the Contribution, v) the inclusion of electronic links from the

For peer review only

Contribution to third party material where-ever it may be located; and, vi) licence any third party to do any or all of the above."

For peer review only

REFERENCES

1. Nepon J, Belik S, Bolton J, Sareen J. The relationship between anxiety disorders and suicide attempts: findings from the National Epidemiologic Survey on Alcohol and Related Conditions. *Depress Anx* 2010;27:791–798, doi: 10.1002/da.20674 [published Online First 9 March 2010].
2. Kessler R, Keller M, Wittchen H. The epidemiology of generalized anxiety disorder. *Psychiatr Clin North Am* 2001;24:19–39, doi: http://dx.doi.org/10.1016/S0193-953X(05)70204-5 [published Online First 29 June 2005].
3. Stein MB, Sareen J. Generalized anxiety disorder. *New Engl J Med* 2015;373:2059–2068, doi: 10.1056/NEJMcp1502514 [published Online First 19 November 2015].
4. Hoge E, Ivkovic A, Fricchione G. Generalized anxiety disorder: diagnosis and treatment. *BMJ* 2012;345,e7500, doi: http://dx.doi.org/10.1136/bmj.e7500 [published Online First 27 November 2012].
5. Shen C, Hu Y, Hu L, et al. The risk of cancer in patients with generalized anxiety disorder: a nationwide population-based study. *PLoS ONE* 2013;8:e57399, doi: 10.1371/journal.pone.0057399 [published Online First 27 February 2013].
6. Roy-Byrne PP, Davidson KW, Kessler RC, et al. Anxiety disorders and comorbid medical illness. *Gen Hosp Psychiatry* 2008;30:208–225, doi: 10.1016/j.genhosppsych.2007.12.006 [published Online First 20 April 2008].
7. Schoevers R, Deeg D, van Tilburg W, Beekman A. Depression and generalized

For peer review only

anxiety disorder: co-occurrence and longitudinal patterns in elderly patients. *Am J Geriatr Psychiatry* 2005;13:31–39, doi: 10.1176/appi.ajgp.13.1.31 [published Online First 24 January 2013].

8. Grant BF, Hasin DS, Stinson FS, et al. Prevalence, correlates, co-morbidity, and comparative disability of DSM-IV generalized anxiety disorder in the USA: results from the National Epidemiologic Survey on Alcohol and Related Conditions. *Psychol Med* 2005;35:1747, doi: 10.1017/S0033291705006069 [published Online First 5 October 2005].
9. The NHS Information Centre for health and social care, Leeds. Adult psychiatric morbidity in England, 2007 Results of a household survey. http://www.esds.ac.uk/doc/6379/mrdoc/pdf/6379research_report.pdf (accessed 29 July 2014).
10. Fryers T, Melzer D, Jenkins R. Social inequalities and the common mental disorders: a systematic review of the evidence. *Soc Psychiatry Psychiatr Epidemiol* 2003;38:229–237, doi: 10.1007/s00127-003-0627-2.
11. Hunt C, Issakidis C, Andrews G. DSM-IV generalized anxiety disorder in the Australian National Survey of Mental Health and Well-Being. *Psychol Med* 2002;32:649–659, doi: 10.1017}S0033291702005512 [published Online First 20 June 2002].
12. Zhang X, Norton J, Carriere I, Ritchie K, Chaudieu I, Ancelin ML. Risk factors for late-onset generalized anxiety disorder: results from a 12-year prospective cohort (the ESPRIT study). *Transl Psychiatry* 2015;5:e536, doi:

For peer review only

10.1038/tp.2015.31 [published Online First 31 March 2015].

13. Subramanian S, Kawachi I. Income inequality and health: what have we learned so far?. *Epidemiol Rev* 2004;26:78–91, doi: 10.1093/epirev/mxh003.

14. Pickett KE, Wilkinson RG. Income inequality and health: a causal review. *Soc Sci Med* 2014;128:316–326, doi: 10.1016/j.socscimed.2014.12.031 [published Online First 30 December 2014].

15. Hayat SA, Luben R, Keevil VL, et al. Cohort profile: a prospective cohort study of objective physical and cognitive capability and visual health in an ageing population of men and women in Norfolk (EPIC-Norfolk 3). *Int J Epidemiol* 2013;1-10 [published Online First 14 June 2013].

16. Surtees P, Wainwright N, Khaw K, Day N. Functional health status, chronic medical conditions and disorders of mood. *Br J Psychiatry* 2003;183:299–303, doi: 10.1192/bjp.183.4.299 [published Online First 30 September 2003].

17. Ware JE, Snow K, Kosinski M, Gandek B. New England Medical Center Hospital. Health Institute. SF-36 Health Survey: Manual and Interpretation Guide. The Health Institute; Boston, MA: New England Medical Center 1993.

18. Elias P, Halstead K, Prandy K. CASOC: Computer-assisted standard occupational coding. London, England: HMSO 1993.

19. Shohaimi S, Luben R, Wareham N, et al. Residential area deprivation predicts smoking habit independently of individual educational level and occupational social class. A cross sectional study in the Norfolk cohort of the European Investigation into Cancer (EPIC-Norfolk). *J Epidemiol Community Health*

For peer review only

2003;57:270–276, doi: 10.1136/jech.57.4.270.

20. Department for Communities and Local Government. English Indices of Deprivation. https://www.gov.uk/government/collections/english-indices-of-deprivation (accessed 15 April 2014).

21. Kessler RC, Duncan GJ, Gennetian LA, et al. Associations of housing mobility interventions for children in high-poverty neighbourhoods with subsequent mental disorders during adolescence. *JAMA* 2014;311:937–948 doi: 10.1001/jama.2014.607 [published Online First 17 June 2016].

22. Poortinga W, Dunstan FD, Fone DL. Perceptions of the neighbourhood environment and self rated health: a multilevel analysis of the Caerphilly Health and Social Needs Study. *BMC Public Health* 2007;7:285 doi: 10.1186/1471-2458–7-285 [published Online First 9 October 2007].

23. Stafford M, Cummins S, Macintyre S, Ellaway A, Marmot M. Gender differences in the associations between health and neighbourhood environment. *Soc Sci Med* 2005;60:1681-1692 doi: 10.1016/j.socscimed.2004.08.028 [published Online First 5 November 2004].

24. Equality and Human Rights Commission. Women, men and part-time work. http://www.equalityhumanrights.com/about-us/devolved-authorities/commission-scotland/legal-work-scotland/articles/women-men-and-part-time-work (accessed June 8, 2015).

25. Pattyn E, Van Praag L, Verhaeghe M, Levecque K, Bracke P. The association between residential area characteristics and mental health outcomes among

For peer review only

men and women in Belgium. *Arch Public Health* 2011;69:3, doi: 10.1186/0778-7367-69-3 [published Online First 24 October 2011].

26. Kavanagh A, Bentley R, Turrell G, Broom D, Subramanian S. Does gender modify associations between self rated health and the social and economic characteristics of local environments?. *J Epidemiol Community Health* 2006;60:490–495, doi: 10.1136/jech.2005.043562.

27. Molinari C Ahern M, Hendryx M. The relationship of community quality to the health of women and men. *Soc Sci Med* 1998;47:1113–1120, doi: http://dx.doi.org/10.1016/S0277-9536(98)00114-2 [published Online First 16 October 1998].

28. Hoehn-Saric R, McLeod D, Funderburk F, Kowalski P. Somatic symptoms and physiologic responses in generalized anxiety disorder and panic disorder. *Arch Gen Psychiatry* 2004;61:913, doi: 10.1001/archpsyc.61.9.913.

29. Chaudieu I, Beluche I, Norton J, Boulenger JP, Ritchie K, Angelin ML. Abnormal reactions to environmental stress in elderly persons with anxiety disorders: evidence from a population study of diurnal cortisol changes. *J Affect Disord* 2008;106:307–313, doi: 10.1016/j.jad.2007.07.025 [published Online First 28 August 2007].

30. Kavanagh AM, Goller JL, King T, Jolley D, Crawford D, Turrell G. Urban area disadvantage and physical activity: a multilevel study in Melbourne, Australia. *J Epidemiol Community Health* 2005;59:934–940, doi: 10.1136/jech.2005.035931.

31. Day A, Livingstone H. Gender differences in perceptions of stressors and

For peer review only

utilization of social support among university students. *Can J Behav Sci* 2003;35:73–83, doi: http://dx.doi.org/10.1037/h0087190.

32. Karno M, Hough R, Burnam M. Lifetime Prevalence of Specific Psychiatric Disorders Among Mexican Americans and Non-Hispanic Whites in Los Angeles. *Arch Gen Psychiatry* 1987;44:695, doi: 10.1001/archpsyc.1987.01800200021004.

33. Peterson BS, Rauh VA, Bansal R, et al. Effects of prenatal exposure to air pollutants (polycyclic aromatic hydrocarbons) on the development of brain white matter, cognition, and behavior in later childhood. *JAMA Psychiatry* 2015;72:531-540 doi: 10.1001/jamapsychiatry.2015.57 [published Online First 1 June 2015].

34. Brauer M. Air pollution, stroke, and anxiety. *BMJ* 2015; 350:h1510 doi: http://dx.doi.org/10.1136/bmj.h1510 [published Online First 24 March 2015].

35. UK Government. Clean Bus Technology Fund 2015. https://www.gov.uk/government/uploads/system/uploads/attachment_data/file/487583/clean-bus-technology-fund-projects-2015.pdf (accessed 2 February 2016).

36. Baxter AJ, Scott KM, Vos T, Whiteford HA. Global prevalence of anxiety disorders: a systematic review and meta-regression. *Psychol Med* 2013;43:897–910 doi: 10.1017/S003329171200147X [published Online First 10 July 2012].

37. Kessler RC, Berglund P, Dmler O, Jin R, Merikangas KR, Walters EE. Lifetime prevalence and age-of-onset distributions of DSM-IV disorders in the National Comorbidity Survey Replication. *Arch Gen Psychiatry* 2005;62:593–602 doi:

For peer review only

10.1001/archpsyc.62.6.593 [published Online First 10 June 2005].

38. Patel V, Araya R, de Lima M, Ludermir A, Todd C. Women, poverty and common mental disorders in four restructuring societies. *Soc Sci Med* 1999;49: doi: 1461–1471 10.1016/S0277-9536(99)00208-7 [published Online First 20 August 1999].

39. Spence SH, Najman JM, Bor W, O'Callaghan MJ, Williams GM. Maternal anxiety and depression, poverty and marital relationship factors during early childhood as predictors of anxiety and depressive symptoms in adolescence. *J Child Psychol Psychiatry* 2002;43:457–69 doi: 10.1111/1469-7610.00037 [published Online First 21 April 2002].

40. Starcevic V. Anxiety disorders in adults: a clinical guide. New York: Oxford University Press; 2005.

41. World Health Organization. Commission on social determinants of health – final report. http://www.who.int/social_determinants/thecommission/finalreport/en/ (accessed 10 July 2014).

42. Godlee F, Walker A. Importance of a healthy environment. BMJ 1991;303:1124-6 [published Online First 2 November 1991].

For peer review only

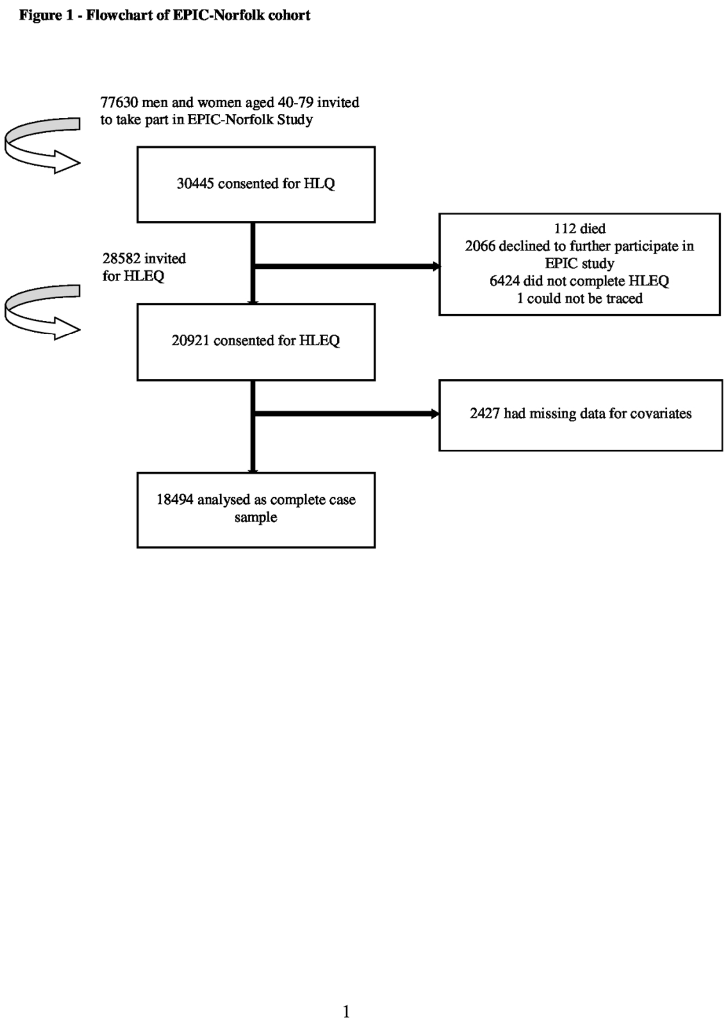

Figure 1 - Flowchart of EPIC-Norfolk cohort

215x279mm (200 x 200 DPI)

Please see the article line numbers (column on the right) and the explanations provided.

STROBE Statement—checklist of items that should be included in reports of observational studies

	Item No	Recommendation	Line numbers within the article
**Title and abstract**	1	(*a*) Indicate the study's design with a commonly used term in the title or the abstract	5, 35
		(*b*) Provide in the abstract an informative and balanced summary of what was done and what was found	27-66
**Introduction**			
Background/rationale	2	Explain the scientific background and rationale for the investigation being reported	85-102
Objectives	3	State specific objectives, including any prespecified hypotheses	104-107, 111-112
**Methods**			
Study design	4	Present key elements of study design early in the paper	116
Setting	5	Describe the setting, locations, and relevant dates, including periods of recruitment, exposure, follow-up, and data collection	114-132
Participants	6	(*a*) *Cohort study*—Give the eligibility criteria, and the sources and methods of selection of participants. Describe methods of follow-up<br>*Case-control study*—Give the eligibility criteria, and the sources and methods of case ascertainment and control selection. Give the rationale for the choice of cases and controls<br>*Cross-sectional study*—Give the eligibility criteria, and the sources and methods of selection of participants	115-118, 123-127, 108-110 (one phrase in the introduction, as well)
		(*b*) *Cohort study*—For matched studies, give matching criteria and number of exposed and unexposed<br>*Case-control study*—For matched studies, give matching criteria and the number of controls per case	
Variables	7	Clearly define all outcomes, exposures, predictors, potential confounders, and effect modifiers. Give diagnostic criteria, if applicable	137-146, 148-169, 171-172, 174-188
Data sources/ measurement	8*	For each variable of interest, give sources of data and details of methods of assessment (measurement). Describe comparability of assessment methods if there is	There were two variables of interest in this study: area deprivation and generalized anxiety disorder (137-

For peer review only - http://bmjopen.bmj.com/site/about/guidelines.xhtml

		more than one group	146, 174-188). The others are potential confounders – in the methods I list them all, indicate how they were assessed and mention that they were collected through the baseline, self-reported postal HLQ questionnaire.
Bias	9	Describe any efforts to address potential sources of bias	215-216
Study size	10	Explain how the study size was arrived at	233-236
Quantitative variables	11	Explain how quantitative variables were handled in the analyses. If applicable, describe which groupings were chosen and why	In the methods, I mention how the variables were derived based on the raw data provided by participants in the questionnaires.<br><br>Dependent variable: 197-198<br>Area-level measures: 206-208<br>Individual-level measures: 214-215 (age), 219-226
Statistical methods	12	(*a*) Describe all statistical methods, including those used to control for confounding	191-231
		(*b*) Describe any methods used to examine subgroups and interactions	Similar to other studies, I conducted analyses separately for men and women. I did not do this because of p-value considerations (statistical significance), but rather, because I felt it was important to do. The literature also shows that women, in general, have a higher risk of anxiety. Also, knowing which gender group is more affected can help with the tailoring of targeted interventions.
		(*c*) Explain how missing data were addressed	This was a complete-case analysis (235).
		(*d*) *Cohort study*—If applicable, explain how loss to follow-up was addressed<br>*Case-control study*—If applicable, explain how matching of cases and controls was addressed<br>*Cross-sectional study*—If applicable, describe analytical methods taking account of sampling strategy	Loss to follow-up was not a problem in this study. We were able to track down all participants using various means, unless they expressed that they wished to be removed from the mailing list (125-127).
		(*e*) Describe any sensitivity analyses	228-231

For peer review only

For peer review only - http://bmjopen.bmj.com/site/about/guidelines.xhtml

**Results**			
Participants	13*	(a) Report numbers of individuals at each stage of study—eg numbers potentially eligible, examined for eligibility, confirmed eligible, included in the study, completing follow-up, and analysed	244-247
		(b) Give reasons for non-participation at each stage	We do not have the reasons for non-participation, because these data were not collected when the study was initiated in 1993. There is some information comparing responders versus non-responders, and the reference given in the methods sections contains further information on this (line 116).
		(c) Consider use of a flow diagram	Flow diagram included in submission.
Descriptive data	14*	(a) Give characteristics of study participants (eg demographic, clinical, social) and information on exposures and potential confounders	253-255 and 268-271; we provided characteristics for those with vs. without GAD, because we felt it was important to show the characteristics of those exposed vs. non-exposed (see also Table 1)
		(b) Indicate number of participants with missing data for each variable of interest	248-250
		(c) *Cohort study*—Summarise follow-up time (eg, average and total amount)	251
Outcome data	15*	*Cohort study*—Report numbers of outcome events or summary measures over time	268-269
		Case-control study—Report numbers in each exposure category, or summary measures of exposure	
		Cross-sectional study—Report numbers of outcome events or summary measures	
Main results	16	(*a*) Give unadjusted estimates and, if applicable, confounder-adjusted estimates and their precision (eg, 95% confidence interval). Make clear which confounders were adjusted for and why they were included	Tables 2 and 3 contains unadjusted and progressively adjusted estimates. In results: 275-295. The reason why these confounders were included is provided in line 171-172. As per strobe, we included this information in the methods (variables included); and we omitted repeating this in the results to reduce redundancy. However, if the editor would like us to repeat this information in the results, we are happy to do so.

For peer review only - http://bmjopen.bmj.com/site/about/guidelines.xhtml

For peer review only

		(*b*) Report category boundaries when continuous variables were categorized	The age cut-offs are provided. In regards to the Townsend index, the methods section states that those below and above the cut-point of zero were compared.
		(*c*) If relevant, consider translating estimates of relative risk into absolute risk for a meaningful time period	
Other analyses	17	Report other analyses done—eg analyses of subgroups and interactions, and sensitivity analyses	311-321 (sensitivity analyses). We repeated the analysis using the IMD, because it is a commonly used index in the UK. Unlike the analysis using the Townsend index (which is longitudinal), the analysis based on the IMD is cross-sectional (limitation).
**Discussion**			
Key results	18	Summarise key results with reference to study objectives	327-334 (especially 333-334 - hypothesis)
Limitations	19	Discuss limitations of the study, taking into account sources of potential bias or imprecision. Discuss both direction and magnitude of any potential bias	388-395
Interpretation	20	Give a cautious overall interpretation of results considering objectives, limitations, multiplicity of analyses, results from similar studies, and other relevant evidence	437-448 (We also have a section comparing our study results to those of others: 416-432, as well as a section on potential mechanisms explaining our findings: 342-375)
Generalisability	21	Discuss the generalisability (external validity) of the study results	394-395
**Other information**			
Funding	22	Give the source of funding and the role of the funders for the present study and, if applicable, for the original study on which the present article is based	466-467

*Give information separately for cases and controls in case-control studies and, if applicable, for exposed and unexposed groups in cohort and cross-sectional studies.

Note: An Explanation and Elaboration article discusses each checklist item and gives methodological background and published examples of transparent reporting. The STROBE checklist is best used in conjunction with this article (freely available on the Web sites of PLoS Medicine at http://www.plosmedicine.org/, Annals of Internal Medicine at http://www.annals.org/, and Epidemiology at http://www.epidem.com/). Information on the STROBE Initiative is available at www.strobe-statement.org.

For peer review only - http://bmjopen.bmj.com/site/about/guidelines.xhtml

BMJ Open

Sex differences in the association between area deprivation and generalised anxiety disorder: British population study

Journal:	*BMJ Open*
Manuscript ID	bmjopen-2016-013590.R1
Article Type:	Research
Date Submitted by the Author:	29-Jan-2017
Complete List of Authors:	Remes, Olivia; University of Cambridge, Department of Public Health and Primary Care<br>Wainwright, Nicholas; University of Cambridge, UK, Department of Public Health and Primary Care<br>Surtees, Paul; University of Cambridge, Department of Public Health and Primary Care<br>LaFortune, Louise; University of Cambridge, Department of Public Health and Primary Care<br>Khaw, Kay-Tee; University of Cambridge, Department of Public Health and Primary Care<br>Brayne, Carol; University of Cambridge, Department of Public Health and Primary Care
**Primary Subject Heading**:	Mental health
Secondary Subject Heading:	Public health, Epidemiology
Keywords:	EPIDEMIOLOGY, MENTAL HEALTH, Anxiety disorders < PSYCHIATRY, PUBLIC HEALTH

SCHOLARONE™
Manuscripts

Sex differences in the association between area deprivation and generalised anxiety disorder: British population study

Olivia Remes[1], Nick Wainwright[2], Paul Surtees[1], Louise Lafortune[1], Kay-Tee Khaw[3], Carol Brayne[1]

[1]Department of Public Health and Primary Care, University of Cambridge, Forvie Site, Robinson Way, Cambridge, UK CB2 0SR

[2](Retired) Department of Public Health and Primary Care, University of Cambridge, 2 Worts' Causeway, Cambridge, UK CB1 8RN

[3]Department of Public Health and Primary Care, University of Cambridge, 2 Worts' Causeway, Cambridge, UK CB1 8RN

Figures: 1; Tables: 3; Appendix: 1

Correspondence to: Olivia Remes or260@medschl.cam.ac.uk, telephone: 0777674550

For peer review only

ABSTRACT

OBJECTIVE

Studies have shown that area-level deprivation measured by factors, such as, non-home ownership, non-car ownership, and household overcrowding, can increase the risk for mental disorders over and above individual-level circumstances, such as, education and social class. Whether area-level deprivation is associated with generalised anxiety disorder independent of personal circumstances, and whether this association is different between British women and men is unknown.

DESIGN

Large, population study.

SETTING

UK population-based cohort.

PARTICIPANTS

30,445 people from the general population aged 40 years and older and living in England consented to participate at study baseline, and of these, 21,000 participants completed a structured health and lifestyle questionnaire used to capture generalised anxiety disorder. Area deprivation was measured in 1991 using Census data, and generalised anxiety disorder was assessed in 1996-2000. 10,275 women and 8,219 men had complete data on all covariates.

MAIN OUTCOME MEASURE

Past-year generalised anxiety disorder defined according to the Diagnostic and Statistical Manual of Mental Disorders, fourth edition (DSM-IV).

RESULTS

In this study, 2.5% (261/10,275) of women and 1.8% (145/8,219) of men had generalised anxiety disorder. Women living in the most deprived areas were over 60% more likely to develop anxiety than those living in areas that were not deprived (OR=1.63, 95%CI: 1.21 to 2.21; p=0.001), but this association between deprivation and generalised anxiety disorder was not apparent in men (OR=1.13, 95% CI: 0.72 to 1.77; p=0.598).

CONCLUSION

The absolute numbers of people living in deprived conditions are large worldwide. This, combined with a growing mental health burden means that the findings obtained in this study remain highly relevant. The World Health Organisation has emphasised the need to reduce social and health inequalities. Our findings provide a strong evidence base to this call, showing that the environment needs to be taken into account when developing

For peer review only

mental health policy; gender is clearly an important factor when it comes to assessing the impacts of the environment, and promoting good mental health.

Key words: Anxiety, anxiety disorders, risk factors, gender

For peer review only

Article summary

Strengths and limitations of this study

- We used a large, population-based sample of middle- and older-aged adults and adjusted for a range of important confounders, such as, sociodemographic factors and medical history.
- We used a structured, self-reported questionnaire to assess presence of past-year GAD, and participants were followed for 7 years.
- We measured area deprivation by employing a commonly-used and theoretically-sound index.
- Those who participated in this study were somewhat less deprived and healthier than individuals living in other parts of England; therefore, our results may not generalize to people living in extremely deprived circumstances.

For peer review only

INTRODUCTION

Generalized anxiety disorder (GAD)[1] is a common and persistent disorder, and is associated with increased risk for disability and suicide.[2–5] GAD can lead to serious impairment in social and occupational functioning, and once it develops, it increases the risk for major depression, substance misuse, and serious physical medical conditions.[5–8] This disorder has a chronic course and is difficult to treat.[5] Consequently, it is important that its risk factors are identified for prevention and targeted intervention.

Few studies have assessed the risk factors of GAD, therefore, evidence-based information is scarce. The studies that have been undertaken have focused on characteristics measured at the level of the individual, such as, personal income and education[9, 10, 11], demographics[12, 13], and family history of psychopathology.[13] However, research has shown that the living context, such as area deprivation, can have profound effects on health, independent of personal characteristics.[14, 15, 16] Area deprivation refers to residential environments or living contexts characterized by factors, such as, high levels of unemployment, non-home ownership, non-car ownership, and low income.[14]

Many studies conducted in western countries have shown that living in areas characterized by high income inequality can lead to significantly increased risks for serious medical conditions and mortality.[15, 16] A meta-analysis of cohort studies including over 60 million participants showed that people living in areas of high income

For peer review only

inequality, as measured by the GINI index, had an increased risk for mortality.[17] Population-based studies further showed that living in disadvantaged neighbourhoods or places where there is high chronic stress can increase the risk for mental disorders, such as, depression.[18, 19, 20]. Whether area deprivation can be used to predict generalized anxiety disorder (GAD) is unknown.

In this population-based, cohort study, we examine the association between area deprivation and generalized anxiety disorder, while controlling for a number of confounders, including previous medical conditions, major depressive disorder, and sociodemographic factors. Results are presented separately for women and men, and this is done for several reasons. Research has shown that women are more likely to develop anxiety compared to men, mainly due to biological, social, and environmental factors.[21, 22, 23] Gender frames access to resources derived from the environment.[21, 22] Compared to men, women have been shown to have less access to material and social conditions, and this can influence mental health. Women also seem to interact with their environment differently. For example, women are exposed to different types of stressors compared to men, because of gender differences in social roles.[18, 21]

Despite these differences, research examining the link between the living context, such as, area deprivation, and mental health from a gendered perspective is scarce. It remains unclear whether there are sex differences in the association between area

For peer review only

deprivation and risk of generalized anxiety disorder – and our objective in this study will be to assess this. Knowing that one sex is at risk of developing anxiety when exposed to deprived circumstances helps to tailor interventions and allocate scarce resources according to need.[24]

For peer review only

METHODS

Data were drawn from the European Prospective Investigation of Cancer (EPIC)-Norfolk, whose design and study methods have been described in detail elsewhere.[25] In brief, a prospective population-based cohort of 30,445 participants ages 40 to 74 years were recruited by post between 1993 and 1997 through general practice age-sex registers in the city of Norwich and the surrounding small towns and rural areas (77,630 people were initially invited to join EPIC-Norfolk). At baseline (1993-97), 30,445 participants consented to join the study and completed a postal Health and Lifestyle (HLQ) questionnaire that captured information on sociodemographics, including age, sex, marital status, highest educational attainment, socio-economic status, and self-reported physician diagnoses of physical diseases. Using participants' postal codes, a measure of area deprivation was derived based on the 1991 Census. Between 1993 and 2000, participants completed self-reported postal questionnaires provided they: 1) were still alive, 2) did not ask to be removed from the study's mailing list, and 3) had a valid mailing address.

During 1996-2000, 20,921 participants completed a structured, psychosocial Health and Life Experiences (HLEQ) questionnaire. During this time, an assessment of generalized anxiety disorder (GAD) and major depressive disorder (MDD) was made according to the Diagnostic and Statistical Manual of Mental Disorders , fourth edition (DSM-IV) [1, 26]. (Figure 1). Using the HLEQ questionnaire, disability measures based on the SF-36 were also derived.[27]

For peer review only

All participants recruited through general-practice registers and who completed a baseline health questionnaire were eligible to be included in our study; those who completed a baseline health check and a psychosocial questionnaire during follow-up were eligible to be included in our analysis.

Dependent variable

The primary outcome in this study was past-year GAD. The self-reported HLEQ questionnaire captured the onset and offset timings of episodes of past-year GAD.[26] Past-year GAD consisted of at least one episode that had offset within 12 months of administration of the HLEQ. DSM-IV GAD was diagnosed if participants reported having uncontrollable, excessive worry for six months or longer on most days than not that resulted in disability or impairment. In addition, at least three of the following symptoms needed to have been present: restlessness, irritability, muscle tension, fatigue, trouble concentrating because of worry, mind going blank, trouble falling asleep, trouble staying asleep, and feeling keyed up or on edge.

Individual-level measures (potential confounders)

Individual-level measures included age, sex, social class, marital status, and educational level. The final categorization of the variables took cell size into account and was also done in accordance with previous literature.[26, 28-33] Social class was derived using the Computer-Assisted Standard Occupational Coding[34] and categorized as follows: I (professionals), II (managerial and technical occupations), III non-manual and III manual

For peer review only

(skilled workers), IV (partly skilled workers), and V (unskilled manual workers). To assign social class to men and women, the male partner's current or past occupation was used. If this information was not available, the female partner's occupation was used. If the social class from either partner was unavailable, then it was coded as missing. The final categorization of social class included manual: skilled manual, partly skilled, and unskilled; and non-manual: professionals, managerial and technical, and skilled non-manual. Marital status was categorized into three groups: married, single (or never married), and others (widowed, divorced, separated). Educational attainment was categorized into high (vocational or formal qualifications at the A- or O-level or degree-level qualifications) vs. low (no formal qualifications).

Individual-level health status was assessed through the construction of a variable capturing major prevalent physical diseases associated with anxiety[35]. This was based on HLQ questions asking participants: "Has the doctor ever told you that you have any of the following?", followed by a list of options, such as allergies, asthma, cancer, stroke, heart attack, diabetes, thyroid conditions, etc. To determine disability levels, we used the physical component summary score (PCS) of the Medical Outcomes Study 36-Item Short Form (SF-36), a widely-used, validated self-assessment tool. A score of 100 represents no disability and 0 represents a high level of disability.[27] PCS scores were dichotomized above and below the median.

For peer review only

All of these individual-level variables were regarded as potential confounders and selected based on the literature and their association with anxiety[35-38] and deprivation[39, 40].

Area-level measure (exposure variable)

To examine area deprivation, we used one of the most commonly-used measures of area deprivation in the UK: the Townsend Index[41, 42]. This index is a composite measure of four variables obtained from the 1991 Census: 1) percentage of economically active residents over age 16 who are unemployed, 2) percentage of households that do not possess a car, 3) percentage of private households that are not owner occupied, and 4) percentage of private households that are overcrowded (have more than 1 person per room). These variables were obtained at the level of the enumeration district. Each variable was standardized by obtaining Z scores (dividing the mean by the standard deviation across enumeration districts in England). The Z values of the four variables were added together to produce a Townsend index score for each enumeration district. Positive values of the index indicate enumeration districts that are more deprived, while negative values indicate those that are less deprived; 0 represents the national mean. The postal codes of participants were record linked to enumeration districts, and participants were considered to live in deprived areas depending on the Townsend index score assigned to their enumeration district.[41]

For peer review only

The Townsend deprivation index was also disaggregated into its four constituent components to determine whether any one of these is associated with GAD or if it is the effect of the combined components that is important.

For peer review only

Statistical analysis

Characteristics of the participants were compared by GAD status. We used correlated data analysis to assess the association between individual- and area-level risk factors of GAD. A population-average model was constructed, which accounted for the potential correlation introduced by the clustering of individuals within enumeration districts. To estimate the population-average effect of the risk factors of interest on past-year GAD, we used generalized estimating equations. As past-year GAD represents a binary outcome (yes/no) and the intra-cluster correlation is assumed to be equal, GEE with a logit link and an exchangeable correlation structure was used. Adjusted odds ratios (OR) and 95% confidence intervals based on robust standard errors were estimated. Standard multivariate logistic regression was also conducted and compared to the findings based on GEE.

Individual-level measures consisted of demographic and socio-economic status variables, whereas the area-level measure comprised the Townsend index. Townsend index scores were used to create a dichotomous variable, with 0 as the cut-point (representing the national average). Similarly, when the Townsend index was disaggregated into its four consistent components, each variable was dichotomized using 0 (the national average) as the cut-point.

Analyses were conducted separately for men and women. First, unadjusted effect estimates were determined. Next, models were constructed to adjust for 1) age, social

For peer review only

class, educational attainment; then for 2) age, social class, educational attainment, lifetime history of MDD; and finally for 3) age, social class, educational attainment, lifetime history of MDD, physical diseases, and disability level. Age was first assessed as a categorical variable, and subsequently divided into 10-year bands. Models were constructed for participants with complete measurements on all covariates. It was not possible to group the GAD variable otherwise since it was created and categorized according to the DSM-IV[26, 43], and area deprivation was analysed in accordance with the literature[31, 44]. In a subsequent analysis, a fully-adjusted model was built in which the Townsend index was replaced by its four constituent components to determine whether any one of these four variables is significantly associated with GAD.

Finally, analyses were run with GAD without MDD as the outcome, in which past-year MDD was excluded. All models used two-sided statistical tests and a p-value of <0.05 was considered statistically significant. Analyses were implemented in Statistical Analysis Software (SAS) Version 9.3 (SAS Institute, Cary, NC).

To arrive at the study size, we went through the following steps: of the 30,445 who completed the baseline HLQ, we retained those participants who completed the HLEQ (20,921), and of these, we kept those people with complete data on all covariates (18,494). (Figure 1)

For peer review only

Patient involvement

There were no patients involved in the development of the research question and outcome measures, the design of the study, or the recruitment to and conduct of the study.

For peer review only

RESULTS

77,630 people from general practices in Norfolk were invited to take part in the study, and of these, 30,445 consented. The characteristics of responders versus non-responders are compared in Appendix I; compared to non-responders, those who took part consisted of slightly more women and slightly younger participants. Of the 30,445 people recruited at baseline, 20,921 completed the HLEQ during follow-up. Of those who completed the HLEQ, 18,494 (88.4%) were available for analysis in this study, because they had data on all covariates. The number of missing observations for each covariate were: 9 for education, 47 for marital status, 497 for GAD, 468 for MDD, 458 for social class, 75 for the Townsend index, and 1386 for the SF-36. Participants were followed between 1993 and 2000 (7 years).

The study sample consisted of a total of 10,275 women and 8,219 men over the age of 40. Table 1 shows the distribution of individual- and area-level characteristics by past-year GAD.

For peer review only

Table 1: Distribution of characteristics for women (n=10,275) and men (n=8,219) who completed the HLEQ questionnaire in the EPIC-Norfolk cohort

	Women		Men	
**Characteristic**	**Number with characteristic**	**Percentage and number with past-year GAD**	**Number with characteristic**	**Percentage and number with past-year GAD**
**Individual-level variables**				
**Socio-demographics**				
**Age (years)**				
<50	1444	3.7 (54)	961	3.2 (31)
50-60	3693	3.2 (119)	2645	2.4 (63)
60-70	3167	1.9 (61)	2739	1.2 (33)
>70	1971	1.4 (27)	1874	1.0 (18)
**Education**‡				
Low	4030	2.1 (83)	2363	1.7 (39)
High	6245	2.9 (178)	5856	1.8 (106)
**Marital status**				
Single	414	3.1 (13)	302	4.0 (12)
Married	7714	2.4 (183)	7221	1.5 (111)
Other*	2147	3.0 (65)	696	3.2 (22)
**Social class**¥				
Manual	3820	2.3 (89)	3281	1.7 (55)
Non-manual	6455	2.7 (172)	4938	1.8 (90)
**Health status**				
**Prevalent physical disease**+				
Yes	5660	3.1 (174)	3836	2.2 (86)
No	4615	1.9 (87)	4383	1.4 (59)
**Disability level**				
High¶	5258	3.3 (172)	4009	2.6 (104)
Low	5017	1.8 (89)	4210	0.97 (41)
**Lifetime MDD**				
Yes	1926	8.7 (167)	934	10.0 (93)
No	8349	1.1 (94)	7285	0.7 (52)
**Area-level variable**				
**Townsend index**				

For peer review only

**Deprivation**				
Yes (>0)	1636	3.9 (64)	1237	2.3 (28)
No (<=0)	8639	2.3 (197)	6982	1.7 (117)

[+] Prevalent physical disease: respiratory disease (asthma and bronchitis), allergies (allergies and hay fever), stroke, heart attack, cancer, diabetes, thyroid conditions, arthritis

[¥] Manual: skilled manual, semi-skilled, non-skilled; non-manual: professionals, managerial, skilled non-manual

[‡] High education: O-level, A-level, degree; low education: refers to no education

[*] Other: divorced, separated, widowed

[¶] Above the median PCS value of 50.6

For peer review only

The overall prevalence of past-year GAD was 2.5% (261/10275) for women and 1.8% (145/8219) for men. Women and men with GAD were less than 50 years of age, of higher educational attainment, single, in non-manual occupations, with prevalent physical diseases, higher levels of disability, and major depressive disorder (MDD) (table 1).

Findings from the correlated data analysis showed that the risk of GAD in women living in the most deprived areas was over 70% higher than in those living in the least deprived areas, even after adjusting for age and socio-economic status (OR=1.77, 95% CI: 1.33–2.36) (table 2).

For peer review only

Table 2: Odds ratios for past-year GAD according to individual- and area-level characteristics for women (n=10,275) who completed the HLEQ questionnaire in the EPIC-Norfolk cohort

	Odds ratios (OR) and 95% CI				
**Characteristic***	**Unadjusted**	**Model A[1]**	**Model B[2]**	**Model C[3]**	**P-value for Model C**
**Individual-level variables**					
**Socio-demographics**					
**Age**					
(per 10 years)	0.65 (0.56–0.74)	0.63 (0.54–0.73)	0.73 (0.62–0.85)	0.66 (0.56–0.77)	<0.0001
**Education‡**					
Low	0.72 (0.55–0.93)	0.85 (0.64–1.12)	0.90 (0.68–1.20)	0.90 (0.68–1.20)	0.475
High	1.00	1.00	1.00	1.00	
**Marital status**					
Single	1.33 (0.75–2.36)	1.31 (0.73–2.36)	1.36 (0.74–2.50)	1.34 (0.73–2.47)	0.348
Married	1.00	1.00	1.00	1.00	
Other*	1.28 (0.96–1.71)	1.48 (1.09–2.00)	1.09 (0.80–1.48)	1.07 (0.79–1.46)	0.671
**Social class¥**					
Manual	0.87 (0.67–1.13)	0.89 (0.68–1.17)	0.89 (0.68–1.18)	0.85 (0.64–1.13)	0.271
Non-manual	1.00	1.00	1.00	1.00	
**Health status**					
**Lifetime MDD**					
Yes	8.34 (6.44–10.79)		7.55 (5.78–9.86)	7.00 (5.34–9.17)	<0.0001
No	1.00		1.00	1.00	
**Prevalent physical disease+**					
Yes	1.65 (1.27–2.14)			1.43 (1.09–1.88)	0.011
No	1.00			1.00	
**Disability level**					
High¶	1.87 (1.45–2.43)			1.88 (1.42–2.49)	<0.0001
Low	1.00			1.00	
**Area-level variable**					
**Townsend index**					

**Deprivation**					
Yes (>0)	1.74 (1.31–2.32)	1.77 (1.33–2.36)	1.65 (1.23–2.22)	1.63 (1.21–2.21)	0.001
No (<=0)	1.00	1.00	1.00	1.00	

1. Adjusted for age, SES (education, marital status, social class)
2. Adjusted for age, SES, lifetime MDD
3. Adjusted for age, SES, lifetime MDD, physical disease and disability

+ Prevalent physical disease: respiratory disease (asthma, bronchitis), allergies (allergies, hay fever), stroke, heart attack, cancer, diabetes, thyroid conditions, arthritis

¥ Manual: skilled manual, semi-skilled, non-skilled; non-manual: professionals, managerial, skilled non-manual

‡ High education: O-level, A-level, degree; low education: refers to no education

* Other: divorced, separated, widowed

¶ Above the median PCS value of 50.6

*The brackets show the reference categories that were used for each categorical variable when it was entered in the models - deprivation: no [ref] vs. yes; GAD: no [ref] vs. yes; education: high [ref] vs. low; marital status: married [ref], single, others; social class: non-manual [ref] vs. manual; lifetime MDD: no [ref] vs. yes; prevalent physical disease: no [ref] vs. yes; disability level: low [ref] vs. high. These reference categories were based on the literature.[26, 28-33] Choosing other groupings for the potential confounders would not have changed the results.

For peer review only

The OR reduced slightly after additionally controlling for major depressive disorder (MDD) (OR=1.65, 95% CI: 1.23–2.22, p=0.001), but remained significant. A strong association was present after further adjusting for prevalent physical diseases and disability (OR=1.63, 95% CI: 1.21–2.21; p=0.001). To further determine the aspect of deprivation that is specifically related to GAD in women, the four separate components of the Townsend index were included in a fully-adjusted model. Results showed that the effect estimates were highest for non-car ownership (OR=1.46, 95% CI: 0.96, 2.23; p=0.080), followed by non-home ownership (OR=1.27, 95% CI: 0.86, 1.88; p=0.237), and were lowest for unemployment (OR=1.07, 95% CI: 0.73, 1.58; p=0.720) and overcrowding (OR=0.75, 95% CI: 0.53, 1.08; p=0.120); these variables did not reach statistical significance.

In men, no association existed between anxiety and area deprivation in both unadjusted and adjusted analyses (model C OR=1.13, 95% CI: 0.72–1.77; p=0.598) (table 3).

For peer review only

Table 3: Odds ratios for past-year GAD according to individual- and area-level characteristics for men (n=8,219) who completed the HLEQ questionnaire in the EPIC-Norfolk cohort

	Odds ratios (OR) and 95% CI				
**Characteristic***	**Unadjusted**	**Model A[1]**	**Model B[2]**	**Model C[3]**	**P-value for Model C**
**Individual-level variables**					
**Socio-demographics**					
**Age**					
(per 10 years)	0.59 (0.49–0.71)	0.58 (0.48–0.71)	0.63 (0.51–0.77)	0.52 (0.41–0.64)	<0.0001
**Education‡**					
Low	0.91 (0.63–1.32)	1.13 (0.75–1.70)	1.16 (0.78–1.74)	1.09 (0.73–1.63)	0.670
High	1.00	1.00	1.00	1.00	
**Marital status**					
Single	2.65 (1.44–4.86)	2.34 (1.26–4.36)	2.67 (1.39–5.10)	2.57 (1.32–5.01)	0.006
Married	1.00	1.00	1.00	1.00	
Other*	2.09 (1.31–3.33)	2.21 (1.39–3.52)	1.48 (0.90–2.44)	1.51 (0.91–2.51)	0.111
**Social class¥**					
Manual	0.92 (0.65–1.29)	0.83 (0.58–1.20)	0.84 (0.58–1.23)	0.74 (0.50–1.09)	0.125
Non-manual	1.00	1.00	1.00	1.00	
**Health status**					
**Life-time MDD**					
Yes	15.38 (10.87–21.76)		14.25 (9.97–20.37)	12.88 (8.99–18.46)	<0.0001
No	1.00		1.00	1.00	
**Prevalent physical disease⁺**					
Yes	1.68 (1.20–2.35)			1.53 (1.07–2.20)	0.021
No	1.00			1.00	
**Disability level**					
High¶	2.71 (1.88–3.90)			3.10 (2.13–4.51)	<0.0001
Low	1.00			1.00	
**Area-level variable**					
**Townsend**					

For peer review only

**index**					
**Deprivation**					
Yes (>0)	1.36 (0.90–2.06)	1.26 (0.82–1.94)	1.19 (0.76–1.85)	1.13 (0.72–1.77)	0.598
No (<=0)	1.00	1.00	1.00	1.00	

1. Adjusted for age, SES (education, marital status, social class)
2. Adjusted for age, SES, lifetime MDD
3. Adjusted for age, SES, lifetime MDD, physical diseases and disability

[+] Prevalent physical disease: respiratory disease (asthma, bronchitis), allergies (allergies, hay fever), stroke, heart attack, cancer, diabetes, thyroid conditions, arthritis

[¥] Manual: skilled manual, semi-skilled, non-skilled; non-manual: professionals, managerial, skilled non-manual

[‡] High education: O-level, A-level, degree; low education: refers to no education

[*] Other: divorced, separated, widowed

[¶] Above the median PCS value of 50.6

*The brackets show the reference categories that were used for each categorical variable when it was entered in the models - deprivation: no [ref] vs. yes; GAD: no [ref] vs. yes; education: high [ref] vs. low; marital status: married [ref], single, others; social class: non-manual [ref] vs. manual; lifetime MDD: no [ref] vs. yes; prevalent physical disease: no [ref] vs. yes; disability level: low [ref] vs. high. These reference categories were based on the literature.[26, 28-33] Choosing other groupings for the potential confounders would not have changed the results.

For peer review only

For peer review only - http://bmjopen.bmj.com/site/about/guidelines.xhtml

We had similar findings when logistic regression was used in these models instead of GEE, suggesting that the intra-class correlation is negligible (findings not shown).

To assess whether deprivation was associated with past-year GAD without MDD in women, we excluded participants reporting past-year MDD (while controlling for all covariates in a fully-adjusted model). Deprivation continued to be strongly associated with past-year GAD (OR= 1.61, 95% CI: 1.06–2.43) (findings not shown). In men, the association was still statistically non-significant (OR=1.34, 95% CI: 0.73–2.47).

For peer review only

DISCUSSION

In this analysis of data from a population-based, cohort study we show, for the first time that area deprivation is significantly associated with increased risk for generalized anxiety disorder (GAD) in women, but not in men. The association in women was independent of characteristics measured at the level of the individual, including socio-demographics and major medical conditions. When we assessed the specific aspects of deprivation associated with anxiety in women, we found that those living in areas characterized by a high level of non-car ownership and non-home ownership were at increased risk of GAD, although the associations were not statistically significant. It appears that it is the overall effect of living in deprivation rather than a particular aspect of the living context that is associated with a statistically significantly increased risk of anxiety in women. It is difficult to confirm causality between area deprivation and GAD; however, a rigorous analysis based on observational data is a reasonable method of examining this relationship. The analysis was rigorous, because we used reliable and commonly-used measures of area deprivation and GAD, controlled for covariates that are associated with the exposure (area deprivation) and outcome (GAD), had access to a large sample size of over 18,000 people, and followed participants for a long period (7 years).

Potential mechanisms

The context as measured by Census composite deprivation indices appears to have a different relationship with the mental health of women and men, even after adjusting for individual socio-economic status, demographics, and other psychiatric and major medical conditions. Several mechanisms can account for this. Women perceive, relate to, and engage differently than men.[45, 46] Women spend more time in their local environment, perhaps due to their greater uptake of part-time work and domestic or child-rearing duties.[47] Since they are more embedded in their local communities, they are also more likely to be exposed to the stress and strain that comes with living in deprived circumstances.[23, 48, 49] Exposure to stress has been associated with central nervous system dysfunction and hypothalamic-pituitary-adrenal (HPA) axis dysregulation, which have been implicated in the etiology of GAD.[50, 51] Women may also perceive the environment differently compared to men. Neighbourhood safety and fear of being assaulted appear to be much more of a concern for women.[48, 52] If women perceive their neighbourhood to be unsafe, they tend to restrict leisure activities, such as walking, and this can have further negative effects for their mental health.[48, 53] Perceiving neighbourhoods as unsafe can also erode social cohesion and can make women more hesitant to create social ties with others.[21] This can increase their risk of depression and related mental disorders, because women derive health benefits from being embedded in social networks.[21] Living in deprivation can also make individuals feel excluded from society and ashamed[54], and these feelings of exclusion are particularly harmful for women's mental health[21, 54].

For peer review only

Men and women may also experience and manifest the effects of stress in different ways.[55] Women who are highly distressed tend to develop internalising disorders, while men are more prone to substance abuse and antisocial personality disorder.[56] The National Epidemiologic Survey on Alcohol and Related Conditions (NESARC) study[57] showed that total number of stressors experienced in life had a significantly stronger association with heavy drinking in men than in women. Therefore, men living in deprivation might be more likely to develop negative outcomes, such as, heavy drinking, rather than anxiety.

Strengths and weaknesses and future research

This study reveals that anxiety in women is strongly linked with area disadvantage. It has several strengths. We had a large, population-based sample of middle- and older-aged adults and adequately adjusted for a range of possible confounders. We used a structured, self-reported questionnaire to assess presence of past-year GAD, and participants were followed for a long period of time. We overcome methodological limitations of previous studies by employing a commonly-used, theoretically-sound measure of area deprivation capturing important features of the environment, such as, unemployment and non-home ownership. We also had a large list of self-reported physician diagnoses of chronic physical diseases that we used to establish medical histories. Despite this, the residual effect of diseases not captured by our study, but that are associated with GAD may be present. Past illness may have been underreported, which may have introduced measurement error and attenuated effect estimates

For peer review only

towards the null. Participants were required to complete detailed dietary and lifestyle questionnaires and undergo periodic health assessments. Because those who participated in EPIC-Norfolk were somewhat less deprived and healthier than individuals living in other parts of England[25], our results may not generalize to people living in extremely deprived circumstances. When comparing the demographic characteristics of responders versus non-responders (Appendix I), we found that participants were slightly younger and slightly more women than men consented. The association found within our cohort is unlikely to be explained by selection bias. It is unlikely that the association in non-responders would be in the opposite direction to that which we obtained in our study.

Another limitation is that some of the areas classified as deprived in 1991 might have shown an improvement in socioeconomic circumstances over time and become more affluent, and vice versa. Although this might present an issue for samples drawn from busy, urban environments, we expect changes in area-level circumstances for the EPIC-Norfolk cohort to have been small. Many EPIC-Norfolk participants come from rural areas, where significant urban development and change in the residential environment are unlikely to have occurred during the study period.[31] Nonetheless, to account for potential changes in GAD rates and area-level circumstances, future studies should assess the association between anxiety and area deprivation at multiple time points.

For peer review only

Although area deprivation was measured in 1991 and GAD in 1996-2000, we expect the association between anxiety and area deprivation in women to be even stronger with more recent data. First, older, as well as, more recent literature has shown that poor women or those living in disadvantage are more likely to develop negative health outcomes, while men less so.[23, 46, 58] Second, women are increasingly taking on multiple roles in society, such as, income-earner, child-bearer, and carer, which is adding to their burden (especially if they are living in deprivation).[24] Third, research has also shown that anxiety rates have been increasing in women in recent times.[59] For these reasons, we expect the association between area deprivation and generalized anxiety disorder to be even stronger in women at the present time.

Future research should consider assessing the risk of GAD in countries with high social and material inequalities, such as the United States, where the rates of anxiety are also some of the highest in the world.[60] Compared to the UK, the overall prevalence of GAD in the US is more than twice as high, and middle-aged people are most affected, with a prevalence of 7.7%.[61] It would be especially informative to repeat this study in less developed parts of the world, such as, India, where poverty is strongly linked to the development of mental disorders, and women's unequal status and social roles in society represent important additional issues.[62]

For peer review only

Implications for future generations and placing our research in context

The consequences of living in deprivation are far-reaching and can affect future generations. Repeated exposure to socioeconomic disadvantage in childhood is a consistent predictor of poor mental health in adolescence and young adulthood, particularly for young girls.[63] Since anxiety disorders tend to emerge in early adolescence, repeated exposure to socioeconomic disadvantage in childhood can increase the risk for more severe, early-onset forms of the disorder. Early-onset forms are the most difficult to treat and have a poor prognosis.[64] Our study is the largest to date to examine the link between area deprivation and generalized anxiety disorder.

Although other studies have shown that the places where people live have a substantial impact on health[15, 16], studies on the links between area deprivation and mental disorders from a gendered perspective are limited. A recent, large, population-based study[18] of over 21,000 people living in Ireland showed that area deprivation was associated with a significantly increased risk for common mental disorders in women, but not in men, after controlling for demographic and socioeconomic factors, and disability. In line with this, a study[21] of over 2700 adults living in Canada showed that greater neighbourhood disadvantage also was associated with increased risk of depressive symptoms in women, but not in men. Research conducted in the US had similar findings.[54] This indicates that characteristics of the living context seem to influence women's health in particular. Very few studies have assessed the association

For peer review only

between deprivation and mental health from a gendered perspective, and research specifically focusing on anxiety disorders is scarcer still.

Our findings differ from the only other population-based, contextual study of generalized anxiety among men and women living in areas of low socioeconomic circumstances.[23] In this cross-sectional study, no association with anxiety was found; however, the measure of deprivation was based only on the local unemployment rate. Thus, the results are not directly comparable to ours. Further, the previous study used the Symptoms Checklist-90-Revised scale to measure *complaints* of generalized anxiety, yielding different estimates than ours. In contrast to the DSM-IV, the Symptoms Checklist-90-Revised scale did not base the definition of generalized anxiety around excessive, uncontrollable worry, which is the central, defining feature of GAD, and used a much shorter time frame to assess symptoms. We used a thorough assessment of DSM-IV GAD, which was measured in the past year. In contrast to the previous study, we also examined area deprivation using common, validated indices, covering a wide range of key domains relating to socio-economic disadvantage, such as, level of crime in an area and access to local health services. Studies assessing other health outcomes have suggested that the residential environment has a larger effect on women's health[23, 46], while individual-level factors relating to social status, such as, employment have the greatest impact on men's health.[45] Among disadvantaged women, it is not low income in itself that leads to poorer health, but rather the inability to derive the necessary resources from the environment to manage daily life; this can

For peer review only

translate into stress and anxiety.[65] Women are becoming financially independent as they enter the labour force, which means that economic hardship now impacts them, as well. Women perceive economic hardship as a barrier to managing daily life and making ends meet, which can increase their anxiety. In contrast, men link joss loss to a decline in social status.[45, 57, 65] When men experience job-related stresses, they tend to externalize the effects of such stress and develop substance abuse.[57, 59]

For peer review only

Interpretation

The absolute numbers of people living in deprived conditions are large worldwide. This, combined with a growing mental health burden means that the findings obtained in this study remain highly relevant. The World Health Organisation[66] has emphasised the need to reduce social and health inequalities. Our findings provide a strong evidence base to this call, showing that "perhaps the most important risks to health are beyond people's immediate control"[67] and that the environment needs to be taken into account when developing mental health policy. Gender is clearly an important factor when it comes to assessing the impacts of the environment, and promoting good mental health. Our study shows that investments made to improve local areas will not benefit all parts of the population equally. Regarding clinical implications, health professionals should consider assessing anxiety in women living in deprivation.

For peer review only

Acknowledgements: OR received a PhD studentship from the National Institute for Health Research.

Competing interest: All authors have completed the ICMJE uniform disclosure form at www.icmje.org/coi_disclosure.pdf and declare: no support from any organisation for the submitted work; no financial relationships with any organizations that might have an interest in the submitted work in the previous three years; no other relationships or activities that could appear to have influenced the submitted work.

Funding: This work was supported by the Medical Research Council UK (grant number SP2024-0201 and SP2024-0204) and Cancer Research UK (grant number G9502233).

Author contributions: OR (corresponding author) had the idea for and conducted the analysis, and wrote the article, along with CB, KK, LL, PS, and NW. All authors provided feedback into the analysis and critically reviewed drafts of the manuscript. All authors have seen and approved the final version. The authors had full access to all the data in the study and take responsibility for the integrity of the data and the accuracy of the data analysis. OR acts as guarantor of the study.

Transparency declaration: OR affirms that the manuscript is an honest, accurate, and transparent account of the study being reported; that no important aspects of the study

For peer review only

have been omitted; and that any discrepancies from the study as planned have been explained.

Role of study sponsors and statement of independence: The funding sources had no role in the design and conduct of the study; collection, management, analysis, and interpretation of the data; and preparation, review, or approval of the manuscript.

Ethical approval: The study has ethics committee approval from Norfolk Ethics Committee (Rec Ref: 98CN01) and all participants gave informed consent.

Data sharing: No additional data available. Original dataset requests should be sent to the corresponding author. Please contact O Remes at or260@medschl.cam.ac.uk for questions about the statistical code.

Copyright: “The Corresponding Author has the right to grant on behalf of all authors and does grant on behalf of all authors, a worldwide licence to the Publishers and its licensees in perpetuity, in all forms, formats and media (whether known now or created in the future), to i) publish, reproduce, distribute, display and store the Contribution, ii) translate the Contribution into other languages, create adaptations, reprints, include within collections and create summaries, extracts and/or, abstracts of the Contribution, iii) create any other derivative work(s) based on the Contribution, iv) to exploit all subsidiary rights in the Contribution, v) the inclusion of electronic links from the

For peer review only

Contribution to third party material where-ever it may be located; and, vi) licence any third party to do any or all of the above."

For peer review only

REFERENCES

1. American Psychiatric Association. (1994). *Diagnostic and Statistical Manual of Mental Disorders* (4th edn). Washington, DC: APA.
2. Nepon J, Belik S, Bolton J, Sareen J. The relationship between anxiety disorders and suicide attempts: findings from the National Epidemiologic Survey on Alcohol and Related Conditions. *Depress Anx* 2010;27:791–798, doi: 10.1002/da.20674 [published Online First 9 March 2010].
3. Kessler R, Keller M, Wittchen H. The epidemiology of generalized anxiety disorder. *Psychiatr Clin North Am* 2001;24:19–39, doi: http://dx.doi.org/10.1016/S0193-953X(05)70204-5 [published Online First 29 June 2005].
4. Stein MB, Sareen J. Generalized anxiety disorder. *New Engl J Med* 2015;373:2059–2068, doi: 10.1056/NEJMcp1502514 [published Online First 19 November 2015].
5. Hoge E, Ivkovic A, Fricchione G. Generalized anxiety disorder: diagnosis and treatment. *BMJ* 2012;345,e7500, doi: http://dx.doi.org/10.1136/bmj.e7500 [published Online First 27 November 2012].
6. Shen C, Hu Y, Hu L, et al. The risk of cancer in patients with generalized anxiety disorder: a nationwide population-based study. *PLoS ONE* 2013;8:e57399, doi: 10.1371/journal.pone.0057399 [published Online First 27 February 2013].
7. Roy-Byrne PP, Davidson KW, Kessler RC, et al. Anxiety disorders and comorbid medical illness. *Gen Hosp Psychiatry* 2008;30:208–225, doi:

For peer review only

10.1016/j.genhosppsych.2007.12.006 [published Online First 20 April 2008].

8. Schoevers R, Deeg D, van Tilburg W, Beekman A. Depression and generalized anxiety disorder: co-occurrence and longitudinal patterns in elderly patients. *Am J Geriatr Psychiatry* 2005;13:31–39, doi: 10.1176/appi.ajgp.13.1.31 [published Online First 24 January 2013].

9. Grant BF, Hasin DS, Stinson FS, et al. Prevalence, correlates, co-morbidity, and comparative disability of DSM-IV generalized anxiety disorder in the USA: results from the National Epidemiologic Survey on Alcohol and Related Conditions. *Psychol Med* 2005;35:1747, doi: 10.1017/S0033291705006069 [published Online First 5 October 2005].

10. The NHS Information Centre for health and social care, Leeds. Adult psychiatric morbidity in England, 2007 Results of a household survey. http://www.esds.ac.uk/doc/6379/mrdoc/pdf/6379research_report.pdf (accessed 29 July 2014).

11. Fryers T, Melzer D, Jenkins R. Social inequalities and the common mental disorders: a systematic review of the evidence. *Soc Psychiatry Psychiatr Epidemiol* 2003;38:229–237, doi: 10.1007/s00127-003-0627-2.

12. Hunt C, Issakidis C, Andrews G. DSM-IV generalized anxiety disorder in the Australian National Survey of Mental Health and Well-Being. *Psychol Med* 2002;32:649–659, doi: 10.1017}S0033291702005512 [published Online First 20 June 2002].

13. Zhang X, Norton J, Carriere I, Ritchie K, Chaudieu I, Ancelin ML. Risk factors for

For peer review only

late-onset generalized anxiety disorder: results from a 12-year prospective cohort (the ESPRIT study). *Transl Psychiatry* 2015;5:e536, doi: 10.1038/tp.2015.31 [published Online First 31 March 2015].

14. National Centre for Research Methods. Townsend deprivation index. http://www.restore.ac.uk/geo-refer/36229dtuks00y19810000.php (accessed 15 April 2014).

15. Subramanian S, Kawachi I. Income inequality and health: what have we learned so far?. *Epidemiol Rev* 2004;26:78–91, doi: 10.1093/epirev/mxh003.

16. Pickett KE, Wilkinson RG. Income inequality and health: a causal review. *Soc Sci Med* 2014;128:316–326, doi: 10.1016/j.socscimed.2014.12.031 [published Online First 30 December 2014].

17. Kondo N, Sembajwe G, Kawachi I, van Dam RM, Subramanian SV, Yamagata Z. Income inequality, mortality, and self rated health: meta-analysis of multilevel studies. *BMJ* 2009;339:b4471 doi: http://dx.doi.org/10.1136/bmj.b4471 [published Online First 11 November 2009].

18. Morrissey K. Gender differences in the association between common mental disorders and regional deprivation in Ireland. *Prof Geogr* 2015;68:129-137 doi: 10.1080/00330124.2015.1054020 [published Online First 24 July 2015].

19. Matheson FI, Moineddin R, Dunn JR, Creatore MI, Gozdyra P, Glazier RH. Urban neighborhoods, chronic stress, gender and depression. *Soc Sci Med* 2006;63:2604-16 doi: http://dx.doi.org/10.1016/j.socscimed.2006.07.001.

For peer review only

20. Galea S, Ahern J, Nandi A, Tracy M, Beard J, Vlahov D. Urban neighborhood poverty and the incidence of depression in a population-based cohort study. *Ann Epidemiol* 2007;17:171-179 doi: 10.1016/j.annepidem.2006.07.008 [published Online First 1 July 2008].

21. Bassett E, Moore S. Gender differences in the social pathways linking neighborhood disadvantage to depressive symptoms in adults. *Plos ONE* 2013;8:e76554 doi: doi:10.1371/journal.pone.0076554 [published Online First 17 October 2013].

22. Kawachi I, Berkman L. Social ties and mental health. *J Urban Health* 2001;78:458-67. doi: 10.1093/jurban/78.3.458.

23. Pattyn E, Van Praag L, Verhaeghe M, Levecque K, Bracke P. The association between residential area characteristics and mental health outcomes among men and women in Belgium. *Arch Public Health* 2011;69:3, doi: 10.1186/0778-7367-69-3 [published Online First 24 October 2011].

24. Remes O, Lafortune L, Khaw K-T, Brayne C. Sex and gender in psychiatry. *Lancet Psychiatry*. In press 2017.

25. Hayat SA, Luben R, Keevil VL, et al. Cohort profile: a prospective cohort study of objective physical and cognitive capability and visual health in an ageing population of men and women in Norfolk (EPIC-Norfolk 3). *Int J Epidemiol* 2013;1-10 [published Online First 14 June 2013].

26. Surtees P, Wainwright N, Khaw K, Day N. Functional health status, chronic medical conditions and disorders of mood. *Br J Psychiatry* 2003;183:299–303,

For peer review only

doi: 10.1192/bjp.183.4.299 [published Online First 30 September 2003].

27. Ware JE, Snow K, Kosinski M, Gandek B. New England Medical Center Hospital. Health Institute. SF-36 Health Survey: Manual and Interpretation Guide. The Health Institute; Boston, MA: New England Medical Center 1993.

28. Leng Y, Wainwright NWJ, Cappuccio FP, et al. Daytime napping and the risk of all-cause and cause-specific mortality: a 13-year follow-up of a British population. *Am J Epidemiol* 2014;179:1115-1124 doi: https://doi.org/10.1093/aje/kwu036 [published Online First 30 March 2014].

29. Leng Y, Wainwright NWJ, Cappuccio FP, et al. Self-reported sleep patterns in a British population cohort. *Sleep Med* 2014;15:295-302 doi: 10.1016/j.sleep.2013.10.015.

30. Leng Y, Wainwright NW, Capppuccio FP, et al. Daytime napping and increased risk of incident respiratory diseases: symptom, marker, or risk factor? *Sleep Med* 2016;23:12-15 doi: 10.1016/j.sleep.2016.06.012 [published Online First 12 July 2016].

31. Shohaimi S, Luben R, Wareham N, et al. Residential area deprivation predicts smoking habit independently of individual educational level and occupational social class. A cross sectional study in the Norfolk cohort of the European Prospective Investigation into Cancer (EPIC-Norfolk). *J Epidemiol Community Health* 2003;57:270-276 doi: 10.1136/jech.57.4.270.

For peer review only

32. Myint PK, Surtees PG, Wainwright NW, et al. Physical health-related quality of life predicts stroke in the EPIC-Norfolk. *Neurology* 2007;69:2243-8 doi: 10.1212/01.wnl.0000296010.21252.78.

33. Mavaddat N, Kinmonth AL, Sanderson S, Surtees P, Bingham, Khaw KT. What determines self-rated health (SRH)? A cross-sectional study of SF-36 domains in the EPIC-Norfolk cohort. *J Epidemiol Community Health* 2011;65:800-806 doi: 10.1136/jech.2009.090845 [published Online First 15 June 2010].

34. Elias P, Halstead K, Prandy K. CASOC: Computer-assisted standard occupational coding. London, England: HMSO 1993.

35. Remes O, Brayne C, Van der Linde R, Lafortune L. A systematic review of reviews on the prevalence of anxiety disorders in adult populations. *Brain Behav* 2016;6:e00497 doi: 10.1002/brb3.497 [published Online First 5 June 2016].

36. Weisberg RB. Overview of generalized anxiety disorder: epidemiology, presentation, and course. *J Clin Psychiatry* 2009;70:4-9. doi: 10.4088/JCP.s.7002.01.

37. Wittchen HU. Generalized anxiety disorder: prevalence, burden, and cost to society. *Depress Anxiety* 2002;16:162-71 doi: 10.1002/da.10065 [published Online First 18 December 2002].

38. Culpepper L. Generalized anxiety disorder and medical illness. J Clin Psychiatry 2009;70:20-4.

39. Lakshman R, McConville A, How S, Flowers J, Wareham N, Cosford P. Association between area level socio-economic deprivation and a cluster of behavioural risk

For peer review only

factors: Cross sectional, population based study. *J Public Health (Oxf)* 2011;33:234-245. doi: 10.1093/pubmed/fdq072 [published Online First 29 September 2010].

40. Vetter S, Endrass J, Schweizer I, Teng H-M, Rossler W, Gallo WT. The effects of economic deprivation on psychological well-being among the working population of Switzerland. *BMC Public Health* 2006;6:223. doi: 10.1186/1471-2458-6-223 [published Online First 4 September 2006].

41. Shohaimi S, Boekholdt MS, Luben R, Wareham NJ, Khaw K-T. Distribution of lipid parameters according to different socio-economic indicators – the EPIC-Norfolk prospective population study. *BMC Public Health* 2014;14:782, doi: 10.1186/1471-2458-14-782 [published Online First 28 August 2014].

42. Townsend P, Phillimore P, Beattie A. Health and Deprivation: Inequalities and the North. London: Croom Helm 1988.

43. Surtees PG, Wainwright NWJ, Brayne C. Psychosocial aetiology of chronic disease: a pragmatic approach to the assessment of lifetime affective morbidity in an EPIC component study. *J Epidemiol Community Health* 2000;54:114-122 doi: 10.1136/jech.54.2.114.

44. Shohaimi S, Welch A, Bingham S, et al. Residential area deprivation predicts fruit and vegetable consumption independently of individual educational level and occupational social class: a cross sectional population study in the Norfolk cohort of the European Prospective Investigation into Cancer (EPIC-Norfolk). *J Epidemiol Community Health* 2004;58:686-691 doi: 10.1136/jech.2003.008490.

For peer review only

For peer review only - http://bmjopen.bmj.com/site/about/guidelines.xhtml

45. Poortinga W, Dunstan FD, Fone DL. Perceptions of the neighbourhood environment and self rated health: a multilevel analysis of the Caerphilly Health and Social Needs Study. *BMC Public Health* 2007;7:285 doi: 10.1186/1471-2458–7-285 [published Online First 9 October 2007].

46. Stafford M, Cummins S, Macintyre S, Ellaway A, Marmot M. Gender differences in the associations between health and neighbourhood environment. *Soc Sci Med* 2005;60:1681-1692 doi: 10.1016/j.socscimed.2004.08.028 [published Online First 5 November 2004].

47. Equality and Human Rights Commission. Women, men and part-time work. http://www.equalityhumanrights.com/about-us/devolved-authorities/commission-scotland/legal-work-scotland/articles/women-men-and-part-time-work (accessed June 8, 2015).

48. Kavanagh A, Bentley R, Turrell G, Broom D, Subramanian S. Does gender modify associations between self rated health and the social and economic characteristics of local environments?. *J Epidemiol Community Health* 2006;60:490–495, doi: 10.1136/jech.2005.043562.

49. Molinari C Ahern M, Hendryx M. The relationship of community quality to the health of women and men. *Soc Sci Med* 1998;47:1113–1120, doi: http://dx.doi.org/10.1016/S0277-9536(98)00114-2 [published Online First 16 October 1998].

50. Hoehn-Saric R, McLeod D, Funderburk F, Kowalski P. Somatic symptoms and physiologic responses in generalized anxiety disorder and panic disorder. *Arch*

For peer review only

Gen Psychiatry 2004;61:913, doi: 10.1001/archpsyc.61.9.913.

51. Chaudieu I, Beluche I, Norton J, Boulenger JP, Ritchie K, Angelin ML. Abnormal reactions to environmental stress in elderly persons with anxiety disorders: evidence from a population study of diurnal cortisol changes. *J Affect Disord* 2008;106:307–313, doi: 10.1016/j.jad.2007.07.025 [published Online First 28 August 2007].
52. Smith WR, Torstensson M, Johansson K. Perceived risk and fear of crime: gender differences in contextual sensitivity. Int Rev Victimol 2001;8:159-181.
53. Kavanagh AM, Goller JL, King T, Jolley D, Crawford D, Turrell G. Urban area disadvantage and physical activity: a multilevel study in Melbourne, Australia. *J Epidemiol Community Health* 2005;59:934–940, doi: 10.1136/jech.2005.035931.
54. Pabayo R, Kawachi I, Gilman S. Income inequality among American states and the incidence of major depression. *J Epidemiol Community Health* 2013; doi: 10.1136/jech-2013-203093 [published Online First 24 September 2013].
55. Day A, Livingstone H. Gender differences in perceptions of stressors and utilization of social support among university students. *Can J Behav Sci* 2003;35:73–83, doi: http://dx.doi.org/10.1037/h0087190.
56. Karno M, Hough R, Burnam M. Lifetime Prevalence of Specific Psychiatric Disorders Among Mexican Americans and Non-Hispanic Whites in Los Angeles. *Arch Gen Psychiatry* 1987;44:695, doi: 10.1001/archpsyc.1987.01800200021004.

For peer review only

57. Dawson DA, Grant BF, Ruan WJ. The association between stress and drinking: modifying effects of gender and vulnerability. *Alcohol Alcohol* 2005;40:453-60 doi: 10.1093/alcalc/agh176 [published Online First 21 June 2005].

58. Kim J, Richardson V, Park B, Park M. A multilevel perspective on gender differences in the relationship between poverty status and depression among older adults in the United States. *J Women Aging* 2013;3:207-226. doi: http://dx.doi.org/10.1080/08952841.2013.795751 [published Online First 14 June 2013].

59. NHS Digital. Adult Psychiatric Morbidity survey. http://content.digital.nhs.uk/catalogue/PUB21748 (accessed 29 September 2016).

60. Baxter AJ, Scott KM, Vos T, Whiteford HA. Global prevalence of anxiety disorders: a systematic review and meta-regression. *Psychol Med* 2013;43:897–910 doi: 10.1017/S003329171200147X [published Online First 10 July 2012].

61. Kessler RC, Berglund P, Dmler O, Jin R, Merikangas KR, Walters EE. Lifetime prevalence and age-of-onset distributions of DSM-IV disorders in the National Comorbidity Survey Replication. *Arch Gen Psychiatry* 2005;62:593–602 doi: 10.1001/archpsyc.62.6.593 [published Online First 10 June 2005].

62. Patel V, Araya R, de Lima M, Ludermir A, Todd C. Women, poverty and common mental disorders in four restructuring societies. *Soc Sci Med* 1999;49: doi: 1461–1471 10.1016/S0277-9536(99)00208-7 [published Online First 20 August 1999].

63. Spence SH, Najman JM, Bor W, O'Callaghan MJ, Williams GM. Maternal anxiety

For peer review only

and depression, poverty and marital relationship factors during early childhood as predictors of anxiety and depressive symptoms in adolescence. *J Child Psychol Psychiatry* 2002;43:457–69 doi: 10.1111/1469-7610.00037 [published Online First 21 April 2002].

64. Starcevic V. Anxiety disorders in adults: a clinical guide. New York: Oxford University Press; 2005.

65. Ahnquist J, Fredlund P, Wamala SP. Is cumulative exposure to economic hardships more hazardous to women's health than men's? A 16-year follow-up study of the Swedish Survey of Living Conditions. *J Epidemiol Community Health* 2007;61:331-6 doi: 10.1093/alcalc/agh176

66. World Health Organization. Commission on social determinants of health – final report. http://www.who.int/social_determinants/thecommission/finalreport/en/ (accessed 10 July 2014).

67. Godlee F, Walker A. Importance of a healthy environment. BMJ 1991;303:1124-6 [published Online First 2 November 1991].

For peer review only

Appendix I: Characteristics of participants who consented (n=30,445) and refused (n=43,452) to take part in the EPIC-Norfolk cohort study

	Percentage (number)	
**Characteristic**	**Consented**	**Did not consent**
**Age**		
<50	27.5 (8366)	33.7 (14647)
50-60	30.3 (9230)	29.5 (12819)
60-70	32.5 (9879)	27.4 (11898)
>=70	9.8 (2970)	9.4 (4088)
**Sex**		
Female	55.0 (16744)	49.0 (21296)
Male	45.0 (13701)	51.0 (22156)

For peer review only

For peer review only - http://bmjopen.bmj.com/site/about/guidelines.xhtml

Figure 1 – Flowchart of EPIC-Norfolk cohort

This is a flowchart showing the number of participants at each study stage: the number approached to participate in the EPIC-Norfolk study, the number enrolled at baseline, and with complete data on all covariates. The EPIC-Norfolk study consists of middle-aged and older British people.

For peer review only

Figure 1 - Flowchart of EPIC-Norfolk cohort

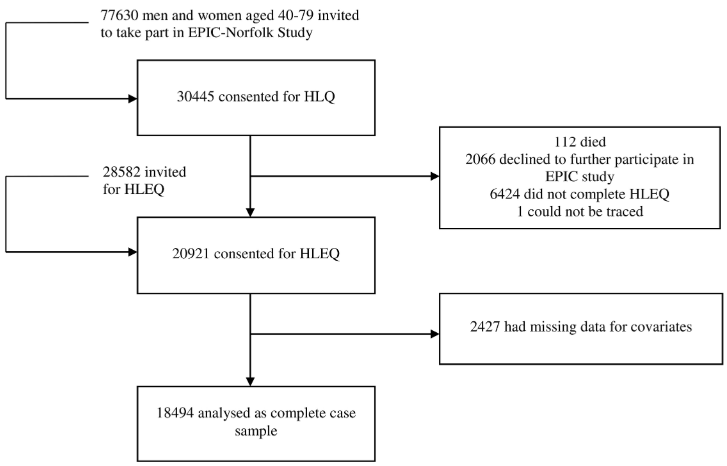

Figure 1 - Flowchart of EPIC-Norfolk cohort

279x361mm (300 x 300 DPI)

Figure 1 - Flowchart of EPIC-Norfolk cohort

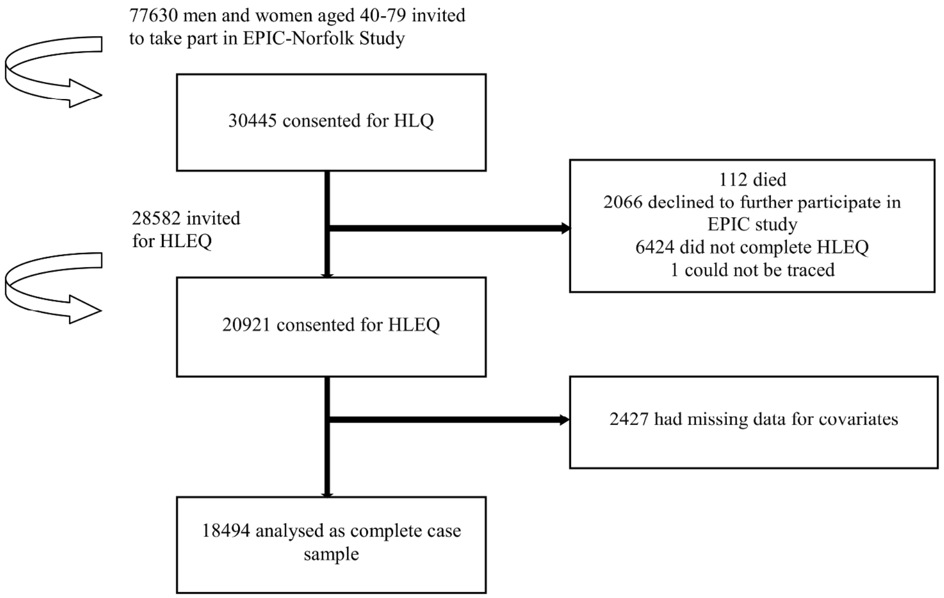

Note to editor: This is the same figure (1), but in a different resolution/style. Please choose whichever one you prefer. They are all at least 300 dpi, as requested.

132x98mm (600 x 600 DPI)

Please see the article line numbers (column on the right) and the explanations provided.

STROBE Statement—checklist of items that should be included in reports of observational studies

	Item No	Recommendation	Line numbers within the article without tracked changes
**Title and abstract**	1	(*a*) Indicate the study's design with a commonly used term in the title or the abstract	5, 39
		(*b*) Provide in the abstract an informative and balanced summary of what was done and what was found	30-62<br>See abstract.
**Introduction**			
Background/rationale	2	Explain the scientific background and rationale for the investigation being reported	91-138<br>See Introduction
Objectives	3	State specific objectives, including any prespecified hypotheses	133-136<br>We included:<br>"In this population-based, cohort study, we examine the association between area deprivation and generalized anxiety disorder, while controlling for a number of confounders, including previous medical conditions, major depressive disorder, and sociodemographic factors."<br>We also add:<br>"It remains unclear whether there are sex differences in the association between area deprivation and risk of generalized anxiety disorder – and our objective in this study will be to assess this."
**Methods**			
Study design	4	Present key elements of study design early in the paper	119<br>We included:<br>"In this population-based, cohort study, we examine…"
Setting	5	Describe the setting, locations, and relevant dates, including periods of recruitment, exposure, follow-up, and data collection	142-157; We reference a study in this paper (reference 25) which contains all of this information.<br>We included the following information in the Methods section:<br>"Data were drawn from the European Prospective Investigation of Cancer (EPIC)-Norfolk, whose design and study methods have been described in detail elsewhere.[25] In brief, a prospective population-based cohort of 30,445 participants ages 40 to 74 years were recruited by post between

For peer review only - http://bmjopen.bmj.com/site/about/guidelines.xhtml

			1993 and 1997 through general practice age-sex registers in the city of Norwich and the surrounding small towns and rural areas (77,630 people were initially invited to join EPIC-Norfolk). At baseline (1993-97), 30,445 participants consented to join the study and completed a postal Health and Lifestyle (HLQ) questionnaire that captured information on sociodemographics, including age, sex, marital status, highest educational attainment, socio-economic status, and self-reported physician diagnoses of physical diseases. Using participants' postal codes, a measure of area deprivation was derived based on the 1991 Census. Between 1993 and 2000, participants completed self-reported postal questionnaires provided they: 1) were still alive, 2) did not ask to be removed from the study's mailing list, and 3) had a valid mailing address.<br><br>During 1996-2000, 20,921 participants completed a structured, psychosocial Health and Life Experiences (HLEQ) questionnaire."
Participants	6	(*a*) *Cohort study*—Give the eligibility criteria, and the sources and methods of selection of participants. Describe methods of follow-up<br>*Case-control study*—Give the eligibility criteria, and the sources and methods of case ascertainment and control selection. Give the rationale for the choice of cases and controls<br>*Cross-sectional study*—Give the eligibility criteria, and the sources and methods of selection of participants	142-148, 152-155, 163-166<br>All of this information is in the Methods section.
		(*b*) *Cohort study*—For matched studies, give matching criteria and number of exposed and unexposed<br>*Case-control study*—For matched studies, give matching criteria and the number of controls per case	
Variables	7	Clearly define all outcomes, exposures, predictors, potential confounders, and effect modifiers. Give diagnostic criteria, if applicable	168-225
Data sources/	8*	For each variable of interest, give sources of data	There were two variables of interest in this study: area deprivation and generalized anxiety disorder

For peer review only - http://bmjopen.bmj.com/site/about/guidelines.xhtml

For peer review only

measurement		and details of methods of assessment (measurement). Describe comparability of assessment methods if there is more than one group	(210-229, 168-177). The others are potential confounders – in the methods I list them all, indicate how they were assessed and mention that they were collected through the baseline, self-reported postal HLQ questionnaire.
Bias	9	Describe any efforts to address potential sources of bias	256-257<br>I mention: "Models were constructed for participants with complete measurements on all covariates."
Study size	10	Explain how the study size was arrived at	269-272<br>We included:<br>"To arrive at the study size, we went through the following steps: of the 30,445 who completed the baseline HLQ, we retained those participants who completed the HLEQ (20,921), and of these, we kept those people with complete data on all covariates (18,494). (Figure 1)"
Quantitative variables	11	Explain how quantitative variables were handled in the analyses. If applicable, describe which groupings were chosen and why	How the variables were handled in analyses (how they were grouped/categorized in preparation for analyses and then how models were constructed with these variables): 181-204, 245-249, 251-256<br><br>Which grouping were chosen and why: As per reviewer 1's comments, I describe the reference categories of the variables in the footnotes under tables 2 and 3. I mention that these groupings were based on the literature and provide the relevant citations.<br><br>How the dependent variable was created: 168-177<br>How area-level measure was created: 210-229<br>Individual-level measures: 179-204; I mention that the final categorization of the variables was also done in accordance with previous literature and I cite the relevant studies.
Statistical methods	12	(*a*) Describe all statistical methods, including those used to control for confounding	231-267 – under Statistical analysis in the paper
		(*b*) Describe any methods used to examine subgroups and interactions	Similar to other studies, I conducted analyses separately for men and women. I did not do this because of p-value considerations (statistical significance), but rather, because I felt it was important to do. In the introduction, I provide the rationale for conducting sex-specific analyses. Knowing which gender group is more affected can help with the tailoring of targeted interventions.
		(*c*) Explain how missing data were addressed	We indicated that this was a complete-case analysis.
		(*d*) *Cohort study*—If applicable, explain how loss to follow-up was addressed	Loss to follow-up was not a problem in this study. We were able to track down all participants using various means, unless they expressed that they wished to be removed from the mailing list.

For peer review only

Case-control study—If applicable, explain how matching of cases and controls was addressed<br>*Cross-sectional study*—If applicable, describe analytical methods taking account of sampling strategy	We elaborate on this in the manuscript.
(*e*) Describe any sensitivity analyses	260-265<br>We included:<br>"In a subsequent analysis, a fully-adjusted model was built in which the Townsend index was replaced by its four constituent components to determine whether any one of these four variables is significantly associated with GAD.<br><br>Finally, analyses were run with GAD without MDD as the outcome, in which past-year MDD was excluded."

For peer review only

For peer review only - http://bmjopen.bmj.com/site/about/guidelines.xhtml

**Results**			
Participants	13*	(a) Report numbers of individuals at each stage of study—eg numbers potentially eligible, examined for eligibility, confirmed eligible, included in the study, completing follow-up, and analysed	435-441<br>We included:<br>"77,630 people from general practices in Norfolk were invited to take part in the study, and of these, 30,445 consented. Of the 30,445 people recruited at baseline, 20,921 completed the HLEQ during follow-up. Of those who completed the HLEQ, 18,494 (88.4%) were available for analysis in this study, because they had data on all covariates. "
		(b) Give reasons for non-participation at each stage	We do not have the reasons for non-participation, because these data were not collected when the study was initiated in 1993. There is some information comparing responders versus non-responders – Appendix I in our paper.
		(c) Consider use of a flow diagram	Flow diagram included in submission.
Descriptive data	14*	(a) Give characteristics of study participants (eg demographic, clinical, social) and information on exposures and potential confounders	291-293, 295-305, 307-311; we provided characteristics for those with vs. without GAD, because we felt it was important to show the characteristics of those exposed vs. non-exposed (see also Table 1)
		(b) Indicate number of participants with missing data for each variable of interest	286-289<br>We included:<br>"The number of missing observations for each covariate were: 9 for education, 47 for marital status, 497 for GAD, 468 for MDD, 458 for social class, 75 for the Townsend index, and 1386 for the SF-36."
		(c) *Cohort study*—Summarise follow-up time (eg, average and total amount)	288-289<br>We included:<br>"Participants were followed between 1993 and 2000 (7 years)."
Outcome data	15*	*Cohort study*—Report numbers of outcome events or summary measures over time	307-308<br>We included:<br>"The overall prevalence of past-year GAD was 2.5% (261/10275) for women and 1.8% (145/8219) for men."
		Case-control study—Report numbers in each exposure category, or summary measures of exposure	
		Cross-sectional study—Report numbers of outcome events or summary measures	

For peer review only - http://bmjopen.bmj.com/site/about/guidelines.xhtml

Main results	16	(*a*) Give unadjusted estimates and, if applicable, confounder-adjusted estimates and their precision (eg, 95% confidence interval). Make clear which confounders were adjusted for and why they were included	Tables 2 and 3 contain unadjusted and progressively adjusted estimates. We also discussed the findings within the text, and provide odds ratios and 95% confidence intervals (313-316, 336-349, 372-376).<br><br>We included the confounders based on the literature – we mention this in the paper and cite relevant literature. As per STROBE, we included this information in the methods section; and we omitted repeating this in the results section to reduce redundancy. However, if the editor would like us to repeat this information in the results, we are happy to do so.
		(*b*) Report category boundaries when continuous variables were categorized	The age cut-offs are provided. In regards to the Townsend index, the methods section states that those below and above the cut-point of zero were compared.
		(*c*) If relevant, consider translating estimates of relative risk into absolute risk for a meaningful time period	
Other analyses	17	Report other analyses done—eg analyses of subgroups and interactions, and sensitivity analyses	372-376, 339-346 (sensitivity analyses)
**Discussion**			
Key results	18	Summarise key results with reference to study objectives	382-386
Limitations	19	Discuss limitations of the study, taking into account sources of potential bias or imprecision. Discuss both direction and magnitude of any potential bias	445-465
Interpretation	20	Give a cautious overall interpretation of results considering objectives, limitations, multiplicity of analyses, results from similar studies, and other relevant evidence	539-550 (We also have a section comparing our study results to those of others: 496-529, as well as a section on potential mechanisms explaining our findings: 403-433)
Generalisability	21	Discuss the generalisability (external validity) of the study results	449-457
**Other information**			
Funding	22	Give the source of funding and the role of the funders for the present study and, if applicable, for the original study on which the present article is based	568-569

For peer review only - http://bmjopen.bmj.com/site/about/guidelines.xhtml

*Give information separately for cases and controls in case-control studies and, if applicable, for exposed and unexposed groups in cohort and cross-sectional studies.

Note: An Explanation and Elaboration article discusses each checklist item and gives methodological background and published examples of transparent reporting. The STROBE checklist is best used in conjunction with this article (freely available on the Web sites of PLoS Medicine at http://www.plosmedicine.org/, Annals of Internal Medicine at http://www.annals.org/, and Epidemiology at http://www.epidem.com/). Information on the STROBE Initiative is available at www.strobe-statement.org.

For peer review only

For peer review only - http://bmjopen.bmj.com/site/about/guidelines.xhtml
